# Internet-Delivered Cognitive Behavioral Therapy for Insomnia Comorbid With Chronic Pain: Randomized Controlled Trial

**DOI:** 10.2196/29258

**Published:** 2022-04-29

**Authors:** Tobias Wiklund, Peter Molander, Philip Lindner, Gerhard Andersson, Björn Gerdle, Elena Dragioti

**Affiliations:** 1 Pain and Rehabilitation Centre, and Department of Health, Medicine and Caring Sciences, Linköping University Linkoping Sweden; 2 Department of Behavioural Sciences and Learning, Linköping University Linköping Sweden; 3 Centre for Psychiatry Research, Department of Clinical Neuroscience, Karolinska Institute, & Stockholm Health Care Services, Region Stockholm Stockholm Sweden; 4 Department of Biomedical and Clinical Sciences, Linköping University Linköping Sweden

**Keywords:** insomnia, chronic pain, comorbid, CBT-i, RCT, web-based CBT, pain, online health, online treatment, digital health, mental health, rehabilitation

## Abstract

**Background:**

Patients with chronic pain often experience insomnia symptoms. Pain initiates, maintains, and exacerbates insomnia symptoms, and vice versa, indicating a complex situation with an additional burden for these patients. Hence, the evaluation of insomnia-related interventions for patients with chronic pain is important.

**Objective:**

This randomized controlled trial examined the effectiveness of internet-based cognitive behavioral therapy for insomnia (ICBT-i) for reducing insomnia severity and other sleep- and pain-related parameters in patients with chronic pain. Participants were recruited from the Swedish Quality Registry for Pain Rehabilitation.

**Methods:**

We included 54 patients (mean age 49.3, SD 12.3 years) who were randomly assigned to the ICBT-i condition and 24 to an active control condition (applied relaxation). Both treatment conditions were delivered via the internet. The Insomnia Severity Index (ISI), a sleep diary, and a battery of anxiety, depression, and pain-related parameter measurements were assessed at baseline, after treatment, and at a 6-month follow-up (only ISI, anxiety, depression, and pain-related parameters). For the ISI and sleep diary, we also recorded weekly measurements during the 5-week treatment. Negative effects were also monitored and reported.

**Results:**

Results showed a significant immediate interaction effect (time by treatment) on the ISI and other sleep parameters, namely, sleep efficiency, sleep onset latency, early morning awakenings, and wake time after sleep onset. Participants in the applied relaxation group reported no significant immediate improvements, but both groups exhibited a time effect for anxiety and depression at the 6-month follow-up. No significant improvements on pain-related parameters were found. At the 6-month follow-up, both the ICBT-i and applied relaxation groups had similar sleep parameters. For both treatment arms, increased stress was the most frequently reported negative effect.

**Conclusions:**

In patients with chronic pain, brief ICBT-i leads to a more rapid decline in insomnia symptoms than does applied relaxation. As these results are unique, further research is needed to investigate the effect of ICBT-i on a larger sample size of people with chronic pain. Using both treatments might lead to an even better outcome in patients with comorbid insomnia and chronic pain.

**Trial Registration:**

ClinicalTrials.gov NCT03425942; https://clinicaltrials.gov/ct2/show/NCT03425942

## Introduction

### Background

Pain conditions are the leading cause of disability and disease burden in the world [1]. In Europe, 19% of the adult population experiences moderate to severe chronic pain [2]. Acute pain is a common symptom of other diseases; however, chronic pain is widely regarded a disease or diseases [3].

Patients with chronic pain often experience insomnia. However, depending on the measures and definitions used, the prevalence rates differ considerably. An earlier study by our group [4] showed that 65% of patients in a specialized pain clinic scored >14 points on the self-report Insomnia Severity Index (ISI), which corresponds to moderate or severe insomnia [5]. Several studies have shown that sleep disturbances increase the risk of developing chronic pain conditions over time [6-8], that poor sleep increases pain levels the next day, and that pain can lead to more insomnia [9-11]. A recent study demonstrated that patients with chronic pain with atypical bedtime habits (12:01 AM to 8:59 PM) experienced higher pain levels, greater activity interference, and higher consumption of prescribed opioids than those with regular bedtime habits (9 PM to midnight) [12]. These results suggest that a misalignment of the circadian rhythm can exacerbate chronic pain.

According to a 2-process model [13], sleep is governed by the circadian rhythm and a homeostatic sleep-wake process. When these processes interact optimally, sleep occurs when we have been awake long enough and it is night (ie, the circadian drive for wakefulness is low). Sleep restriction therapy is a common component of cognitive behavioral therapy for insomnia (CBT-i) and is hypothesized to target these 2 processes [14,15]. The American Academy of Sleep Medicine recommends using CBT-i as the first-line intervention not only for chronic insomnia but also for comorbid insomnia [16]. Several studies have shown that CBT-i is efficacious for treating insomnia co-occurring with chronic pain [17,18].

Typically, CBT-i consists of several components such as a sleep diary, stimulus control, sleep restriction, and advice regarding sleep hygiene [19]. Some treatment manuals also include relaxation training and cognitive interventions to cope with worry, maladaptive thoughts, and depressive symptoms [19]. In addition, cognitive interventions such as cognitive restructuring were not included because the knowledge about what maladaptive thoughts are specific to patients with chronic pain is lacking [20-22]. No consensus exists regarding the components needed for successful treatment; however, the use of stimulus control or sleep restriction predicts outcomes [23]. As stimulus control is based on classical conditioning, patients are told that the bedroom should only be used for sleep or sex to associate the bed with sleep rather than wakefulness. Patients are also instructed to leave the bedroom if they do not fall asleep within a predefined period, usually between 15 and 20 minutes.

Because access to therapists offering CBT-i is limited, internet-delivered treatments are a promising alternative [24]. Standardized internet treatments could potentially increase the availability of nonpharmacological insomnia treatment in primary care as well as in specialized pain clinics. Open-source and evidence-based treatments will improve care by addressing a common aspect of chronic pain, that is, insomnia symptoms. In addition, the implementation of internet-based cognitive behavioral therapy for insomnia (ICBT-i) has immense potential in terms of cost-effectiveness, as the time per patient is shorter than face-to-face CBT-i [25,26]. Previous work from members of our group on persons with insomnia disorder showed that CBT-i provided via the internet (ICBT-i) has comparable outcomes to cognitive behavioral therapy (CBT) provided in a group setting [27]. ICBT-i can take various forms with respect to treatment content and therapist support. Today, there are several studies on fully atomized treatments based on artificial intelligence [28,29]. It has been demonstrated that therapist support, usually via written messages, increases the effect of CBT-i [30]. However, there is no available data on ICBT-i targeting insomnia symptoms in patients with chronic pain.

### Objectives

This randomized controlled trial (RCT) investigates the acceptability of an ICBT-i treatment and assesses whether ICBT-i is more effective than internet-administrated applied relaxation (active control condition) in reducing insomnia symptoms (as measured by the ISI) comorbid to chronic pain. In addition, this RCT investigates the effects of ICBT-i on sleep diary measures, pain intensity, anxiety, depression, pain-related disability, and perceived health. Negative effects were also monitored and reported. Our primary hypothesis is that compared with an active control condition, ICBT-i would lead to greater reductions in insomnia symptoms at the end of treatment. We also hypothesized that ICBT-i would be superior to an active control condition in terms of improvement in symptom-related sleep diary measures, pain intensity, anxiety, depression, pain-related disability, and perceived health. In addition, the improvements gained from treatment are hypothesized to be maintained for 6 months after treatment.

## Methods

### Design

#### Participants and Study Procedure

This randomized controlled parallel-group study was conducted in Sweden and followed the CONSORT (Consolidated Standards of Reporting Trials) eHealth Checklist (Multimedia Appendix 1). The trial was registered at ClinicalTrials.gov (NCT03425942). Participants were selected via the Swedish Quality Registry for Pain Rehabilitation, a survey distributed during all first visits at the Pain and Rehabilitation Centre, Linköping University Hospital, Sweden. The Swedish Quality Registry for Pain Rehabilitation contains data on pain (intensity, duration, and spreading), psychological strain, and the ISI. An estimated effect size of 0.6 and an α of .05, gave a sample of at least 40 participants per study condition [27,31]. An anticipated dropout of one-third resulted in the expected need for a total sample of 120 participants.

Participants with an ISI score of >14 (moderate to severe insomnia symptoms) and aged 18-65 years were asked to participate in the trial either by their physician or via postal invitation. Retrospective analyses were performed to identify former patients with an ISI score of >14 over the previous 2 years. If interested, participants registered on the website and completed another survey covering anxiety and depressive symptoms, demographic and physiological variables, current insomnia symptoms, pain characteristics, pain-related disability, and perceived health. Again, a cutoff score of >14 points on the ISI was used as an inclusion criterion. This measurement was considered as baseline. Participants were also asked to rate their average pain intensity during the previous week on a numeric rating scale as part of the baseline assessment. Finally, they were asked, “For how long have you had your pain problem?” The following predefined answers were provided: (1) less than 3 months, (2) 3 months to 1 year, (3) 1 to 3 years, (4) 3 to 5 years, or (5) >5 years. Participants who gave responses (2) to (5) were included in the study. When this strategy had been applied, the study sample consisted of 54 participants, and the decision was made to end recruitment and analyze that smaller sample.

When informed consent was provided, a staff member called the participant to set up a telephone interview within 5 days from registration on the website. The interview included the Mini International Neuropsychiatric Interview [32] (Swedish translation version 7.0.0) to cover psychiatric comorbidity and to identify bipolar disorder and psychoses, which were the absolute exclusion criteria. In addition, the telephone interview was used to evaluate whether the participant fulfilled the diagnostic criteria for insomnia disorder according to the Diagnostic and Statistical Manual of Mental Disorders, 5th Edition (Table S1 in Multimedia Appendix 2) [33] and to assess other inclusion and exclusion criteria. These criteria are detailed in Table S2 in Multimedia Appendix 2. All cases were evaluated by an inclusion and exclusion committee consisting of 2 experienced psychologists and 1 psychiatrist, when required.

Additional telephone interviews were conducted after treatment and after 6 months to evaluate the fulfillment of the criteria for insomnia disorder (Table S2 in Multimedia Appendix 2).

#### Randomization and Blinding

Included participants were randomized via random.org in blocks of 34 participants to either the ICBT-i group or the applied relaxation group. This procedure was performed to ensure equal numbers of participants in each arm after the first postal survey. Thereafter, participants were added to a randomized list consecutively (ensuring an equal sample size after 34 additional participants). Participants were blinded to the experiment or control and were told that they were assigned to 1 of the 2 treatments. Allocation was conducted after the inclusion and exclusion committee met, and participants were notified the week before treatment started. The therapists were not blinded.

### Ethics Approval and Consent to Participate

The study was approved by the ethical review board in Linköping (dnr. 2014/191-31). An additional application to the ethical committee was submitted (dnr. 2017/511-32) to enable invitation of patients further back in time. The participants also provided informed consent as directed by the World Medical Association Declaration of Helsinki.

### Interventions

The description of the activities per session for both interventions is provided in Table S3 in Multimedia Appendix 2. Both treatment arms were provided with weekly home assignments. The rationale for the interventions and information about sleep were provided via SMS text messaging or PowerPoint (Microsoft Corp) presentations with speaker voice; participants were free to choose modality based on preference. Treatments were always initiated on Mondays, as the participants were gaining access to a new treatment module. They were instructed to hand in their home assignments the next Sunday to receive feedback or therapist support within 48 hours. Handing in-home assignments was mandatory to gain access to the next treatment module, and no treatment lasted longer than the agreed 5 weeks.

### Experimental Group

The ICBT-i treatment conducted is a novel treatment developed by TW and PM and provided via the internet platform iterapi.se [34]. The treatment is based on the most well-established CBT principles for the treatment of insomnia (ie, sleep restriction and stimulus control) [35,36]. The first week focused on a short treatment rationale and registration of current sleep patterns (sleep diary). On the basis of the sleep diary data, an individually designed sleep prescription (bedtime) was calculated and applied during the second week. The prescription was equal to the calculated average total sleep time, but no shorter than 4 hours a night. The prescribed sleep time was maintained for the rest of the treatment unless the sleep efficiency exceeded 85% or sunk below 75%; then, the prescribed sleep time was adjusted to 15 to 30 minutes a week. High sleep efficiency resulted in more time in bed and low sleep efficiency resulted in less time in bed (although not <4 hours/night). The third module taught stimulus control, which is based on classical conditioning. Participants were told to use the bedroom only for sleep and sex. That is, activities such as watching television, reading, and social media consumption were not to be conducted in the bedroom. In addition, the participants were told to go to bed only when sleepy and to get out of bed and leave the bedroom when unable to sleep. Similarly, the participants were told not to sleep in places other than the bedroom or outside the prescribed bedtimes. If stimulus control is implemented, it will restrict time in bed, similar to sleep restriction.

Week 4 was dedicated to daytime activity. Advice regarding activity balance was provided. This advice is based on the research conducted by Andrews et al [37], who found that patients with chronic pain who engage in irregular daytime activities experience poor sleep the same night. Daytime sleepiness can lead to inactivity; however, excessive activity can increase pain and hinder the ability to wind down before bedtime. The advice aims to facilitate a healthy level of daytime activity to promote sleep and create a contrast between daytime activities and nighttime rest. The last week focused on maintaining behavior changes and preventing relapse. Generally, the aim is to keep the intervention as brief as possible while maintaining the main part of the treatment effect. Therefore, some usual CBT-i components were excluded, such as behavioral activation, scheduled worry time, and cognitive restructuring.

### Applied Relaxation Control Condition

The internet-based control condition is a slightly modified version of the well-established applied relaxation techniques developed by Öst [38]. This method was chosen because it is a common treatment component that has a credible and applicable rationale for the treatment of both insomnia [39,40] and chronic pain [41,42]. Furthermore, applied relaxation is an active and rather time-consuming treatment that is supposed to control for time spent, measurement effects, and therapist support. The manual was adapted so that the length of treatment matched that of the experimental group (5 weeks). In addition to a short rationale and registration of current sleep patterns (sleep diary), the first week focused on progressive relaxation and diaphragmatic breathing. Participants were told to practice for 15 minutes twice a day and to keep a log for registration and evaluation. In the following week, time was shortened to 7 minutes twice a day. During the third week, participants were taught conditioned relaxation, and exercises were 2 to 3 minutes long. Differentiated relaxation aims only to activate the muscles required to perform a specific task (ie, other muscles can be relaxed). This was the focus of the fourth week of the study. During the fifth week, participants were taught quick relaxation. As this technique can result in relaxation in just a few seconds, it can be applied several times throughout the day and at bedtime.

### Therapist Support

Both treatment arms had therapist support every week of treatment. Support was provided via written messages in the treatment platform. Therapists (master’s students in psychology or senior psychologists) provided problem solving and feedback on weekly tasks and ensured the correct implementation of treatment components. Because therapist support is one of the factors that contribute to treatment outcome [30], there was no restriction of therapist support, as it did not include components from the other treatment arm. The master’s students were supervised by senior psychologists trained in CBT (TW and PM). In a few cases, telephone calls were used to solve technical problems or to reach participants who did not respond to written messages. The same therapists provided both treatments, and the participants were distributed because of the randomization. Because the treatment content was standardized in both treatment arms, no measures of therapist fidelity were collected.

### Demographic and Physiological Variables

Age, sex, height (in cm), weight (in kg), and educational level were recorded at baseline. The duration of pain and sleep problems were measured using two questions: How long have you had pain problems? How long have you had sleep problems? For pain problems, five predefined answers were provided: <3 months, 3 months to 1 year, 1 to 3 years, 3 to 5 years, and >5 years. For sleep problems, six predefined answers were provided: <1 month, 1 to 3 months, 3 months to 1 year, 1 to 3 years, 3 to 5 years, and >5 years. Finally, participants were asked about the debut of pain or sleep problems: the pain problems preceded the sleep problems, the sleep problems preceded the pain problems, both arose at the same time, and they did not know.

### Primary Outcome: Insomnia Severity

The Swedish version of the ISI was used to quantify perceived insomnia severity. The ISI, a valid instrument with excellent internal consistency, captured the severity and impact of insomnia symptoms [5,43]. The psychometric properties of the Swedish version have also been evaluated in patients with chronic pain [44]. Cronbach α for the ISI in our sample was .67. The ISI is rated on a 5-point Likert scale (0-4) and has a maximum total score of 28. In this study, the ISI was measured at baseline, at the end of every week during treatment (T_1_-T_5_), and at the 6-month follow-up. Morin et al [5] suggested that a minimally important difference of >7 points be used in treatment studies.

### Secondary Outcomes

#### Sleep Parameters Assessed by Responsive Sleep Diary

All participants were asked to complete a sleep diary throughout the treatment and for 1 week at the 6-month follow-up. These data were used to calculate the sleep-specific outcomes, that is, sleep onset latency (SOL), wake time after sleep onset (WASO), time in bed, sleep efficiency, and early morning awakenings (EMA), according to Buysse et al [45]. Every measure is based on the mean value form all registered nights that week. The patient interface presents the time in bed, total sleep time, and sleep efficiency for every night and the mean values for every week.

#### Sleepiness

Sleepiness was measured using the Karolinska Sleepiness Scale (KSS), which is a 9-graded scale that measures the level of sleepiness at a particular time during the day [46]. Every other step is labeled as follows: 1=very alert, 3=alert, 5=neither alert nor sleepy, 7=sleepy (but not fighting sleep), and 9=very sleepy (fighting sleep). The KSS is correlated with several electroencephalogram measures (eg, α-activity, *r*=0.40) and behavior variables (eg, mean reaction time on the psychomotor vigilance task, *r*=0.57) related to sleepiness. As test-retest reliability depends on the time of day, the KSS was sent to participants via SMS text messaging at 11 AM every day during treatment. If the participants did not answer the question within 60 minutes of receiving the SMS text message, the value was reported as missing. The KSS was added to the sleep diary to capture nonrestorative sleep and daytime symptoms.

#### Anxiety

The Generalized Anxiety Disorder–7-item (GAD-7) scale was used to measure anxiety symptoms [47]. The GAD-7 measures symptoms of generalized anxiety disorder but is also highly correlated with more general anxiety measures [47]. Each item has an answer response ranging from 0 to 3 and is scored with respect to the frequency of the symptom during the previous 2 weeks: not at all, several days, more than half the days, and nearly every day. This gives a total score of 0 to 21. Optimal sensitivity/specificity ratio for detecting generalized anxiety disorder is obtained with a cutoff score of ≥10. The GAD-7 has shown excellent internal consistency (Cronbach α=.92) [47]. The Cronbach α for the GAD-7 in our sample was .88.

#### Depression

The Patient Health Questionnaire–9 items (PHQ-9) was used to measure depression [48].

The PHQ-9 is a 9-item self-rating scale reflecting the Diagnostic and Statistical Manual of Mental Disorders, 4th Edition, criteria for major depressive disorder [48]. Each item ranges from 0 to 3 points and is scored with respect to the frequency of the symptom during the last 2 weeks: not at all, several days, more than half the days, and nearly every day. This gives a total score of 0 to 27. The PHQ-9 has shown excellent internal consistency (Cronbach α=.86-.89) [48] and strong correlations with other well-established depression scales such as the Beck Depression Inventory II (*r*=0.84) and the Montgomery-Åsberg Depression Rating Scale (*r=*0.79) [49]. The Cronbach α for the PHQ-9 in our sample was .82. Optimal sensitivity/specificity ratio for detecting depression is obtained with a cutoff score of ≥10.

#### Pain Intensity

The participants were asked to rate their average pain intensity during the previous week on a numeric rating scale ranging from 0 (no pain) to 10 (worst imaginable pain). This scale has provided good validity in experimental conditions [50].

#### Number of Anatomical Pain Regions

The participants were presented with a list of 36 anatomical regions covering the entire body and were asked to mark all regions where they experienced pain. A total score index was calculated (total score=0-36) [51].

#### Pain Disability

The Pain Disability Index (PDI) was used to measure the self-reported pain-related disability [52]. The PDI has high internal consistency (Cronbach α=.85) in patients seeking specialized pain care [53]. Minimal important change depends on baseline scores [54] (eg, baseline values 28-42 require a decrease of at least 15 points). The Cronbach α for the PDI in our sample was .81.

#### Health-Related Quality of Life

The Health Visual Analog Scale from the European Quality of Life instrument was used to measure the current state of self-estimated health [55]. The item consists of a 100-point thermometer-like vertical scale with defined end points (worst imaginable health condition and best imaginable health condition). Higher values indicate better health, whereas lower values indicate worse health [55,56].

#### Potentially Adverse and Unwanted Events

The Negative Effects Questionnaire (NEQ), used to assess the side effects of psychological treatment, consists of 32 items with three parts [57]: an initial yes or no question regarding the occurrence of the negative effect in question and a rating of the impact of the negative effect using a 5-point scale (not at all to extremely). The patient also judges whether the negative effect was caused by the treatment. In this RCT, the NEQ was used to screen for and quantify the most common negative effects attributed to the treatment. There is no consensus in the scientific community on how to present results for negative effects, as definitions and measures vary. Generally, it is recommended to provide the frequencies of the negative effects that have occurred the most. The NEQ has good internal consistency (α=.95) and six factors: symptoms, quality, dependency, stigma, hopelessness, and failure [58].

The Cronbach α for the NEQ in our sample was .80.

#### Compliance

The number of treatment modules with submitted tasks or worksheets was reported as median values and their IQR. Modules with submitted tasks or worksheets were used as proxies for treatment compliance. This definition did not require full completion of all tasks or worksheets of a module, and any answer (in addition to a sleep diary) was interpreted as a result of working with the treatment content from that module.

#### Credibility

Credibility and expectations were measured several times to evaluate the design of the single-blinded control condition. At week 3, the participants answered the following question: to what extent do you think this treatment will be helpful in reducing your sleep problems? The participants answered the question on a 6-point scale with the following end points: the treatment will not help at all and the treatment will help to a large extent. At week 5 and follow-up, the participants answered the following question: how likely is it that you would recommend this treatment to a relative or friend with the same kind of problem? The participants answered the question on a 6-point scale with the following end points: not likely at all and very likely. At follow-up, the participants also answered the following question: to what extent do you think this treatment has been helpful in reducing your sleep problems? The participants answered the question on a 6-point scale with the following end points: the treatment has not helped at all and the treatment has helped to a large extent. The results were also reported as median values and IQR.

### Statistics

The analysis was based on the intention-to-treat principles and the assumption that data were missing at random. Data processing and statistics were performed using the statistical package IBM SPSS Statistics (version 26.0; IBM Corp), the software R (version 4.0.3), and JAMOVI (version 1.2.27). In addition, 2-sided statistical tests were used, and a *P*<.05 was considered significant. Average weekly scores for the sleep diary parameters were calculated using R software. Minor corrections were applied to the sleep diary data to account for missing data and input errors: missing durations (but not timestamps) for otherwise filled entries were replaced with zeros; bed and sleep time were ordered so that the former always came before the latter; *k*=9 entries with negative sleep time (primarily due to mathematically impossible wake durations) were omitted; and *k*=8 entries obviously incorrectly inputted as timestamps were manually recoded as durations.

For descriptive analysis, we used mean values with SDs or median with IQR for continuous variables after normality testing and number with percentage (n, %) for categorical variables. Whenever feasible, owing to the different time point measurements across outcomes, mean changes for both primary and secondary outcomes were applied before and after treatment and 6-month follow-up. For example, for the sleep diary and KSS measurements, preassessments were not available.

To examine the immediate (week 5) and long-term (6-month follow-up) treatment effects on the ISI and secondary outcomes, separate mixed models for repeated measures with treatment, time, and the interaction of treatment by time were performed with random intercept and random slope (whenever appropriate) using an unstructured covariance matrix. The examination of the variance of the slope for time was significant; therefore, a model with random slopes for time with multiple time points fitted the data, and a model with fixed slopes was more appropriate for time with 2 time points. Time was modeled as a numeric variable (0-4 and 0-1), and both linear and quadratic trajectories were considered; the latter was chosen when modeling revealed a significant trajectory. The Akaike information criterion was used to assess model fit: the smaller the Akaike information criterion value, the better the model fit [59]. All available time points were included in the models. On the basis of the principle that data were missing at random, all linear mixed models were run with restricted maximum likelihood estimation, which can produce unbiased estimators [60].

## Results

### Descriptive Results

**Figure 1:**
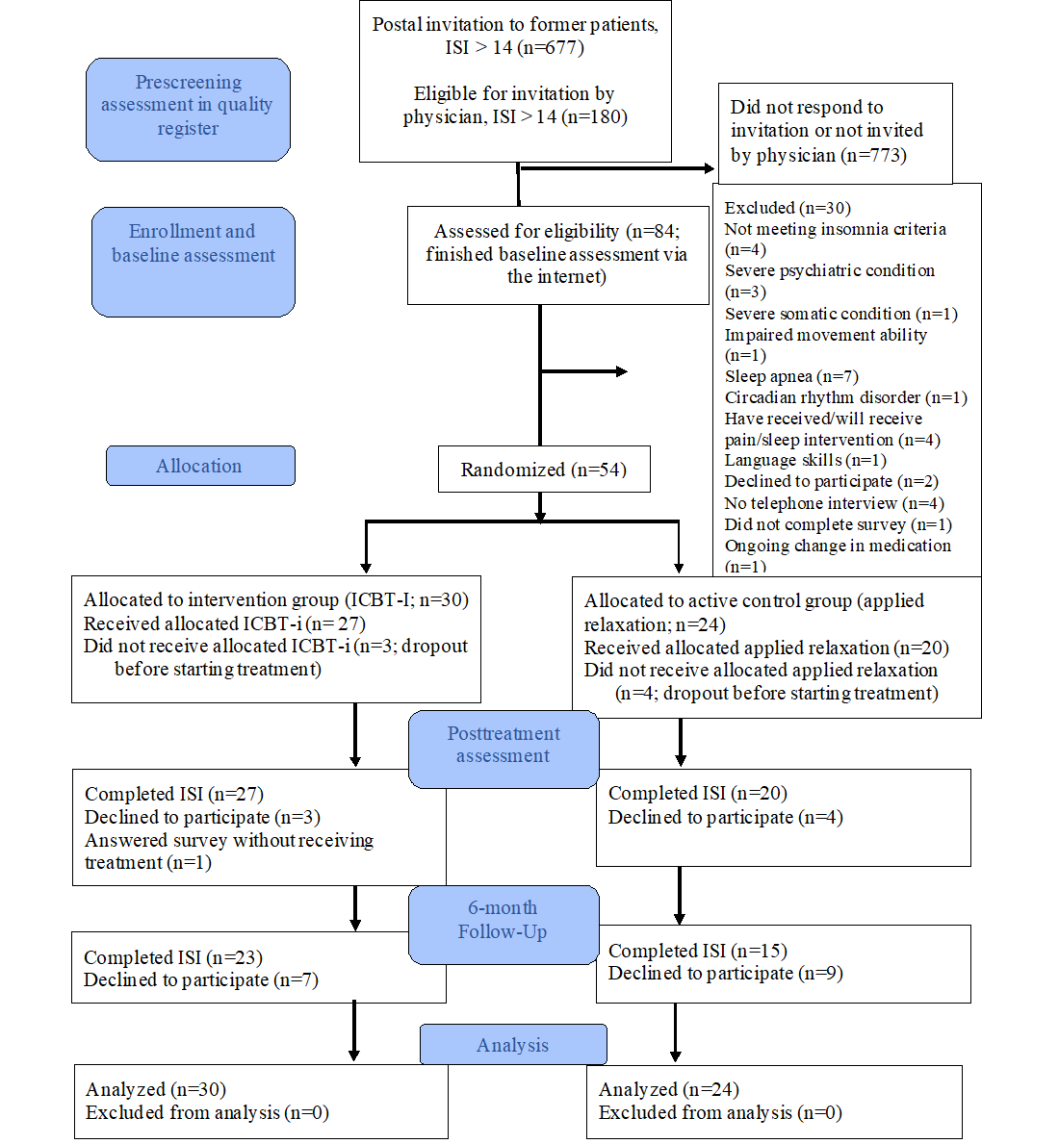
CONSORT (Consolidated Standards of Reporting Trials) 2010 flow diagram. ICBT-i: internet-based cognitive behavioral therapy for insomnia; ISI: Insomnia Severity Index.

Of the 677 former patients who received postal invitations, 188 (27.8%) participants were identified as eligible at their first visit. Of these 188 participants, 86 (45.7%) completed the web-based assessment, and 54 (28.7%) were included and randomized (Figure 1). Table 1 presents the characteristics of the sample. The mean age for the total sample was 49.3 (SD 12.3; range 21-67) years, 37% (20/54) had a university degree, and most participants were women (45/54, 83%). Until this point, no participant had been aware of their allocated intervention (ICBT-i or applied relaxation). Eventually, 30 participants were allocated to the ICBT-i group and 24 to the applied relaxation group. The mean age for the ICBT-i group was 48.2 (SD 11.1; range 27-66) years, and that for the applied relaxation group was 50.6 (SD 13.6; range 21-67) years. At the 6-month follow-up, 77% (23/30) of the participants in the ICBT-i group and 63% (15/24) of the participants in applied relaxation group (ie, the control group) responded.

**Table 1:** Baseline characteristics of the study sample.

Characteristics	ICBT-i^a^ (n=30)	AR^b^ (n=24)	Total (N=54)
Age (years), mean (SD)	48.2 (11.1)	50.6 (13.6)	49.3 (12.3)
Female, n (%)	23 (77)	22 (92)	45 (83)
BMI, mean (SD)	27.0 (5.3)	24.5 (5.2)	25.9 (5.4)
University education, n (%)	12 (40)	8 (33)	20 (37)
ISI^c^, mean (SD)	21.4 (3.3)	20.3 (2.8)	20.9 (3.1)
GAD-7^d^, mean (SD)	7.4 (4.2)	8.3 (5.4)	7.8 (4.7)
PHQ-9^e^, mean (SD)	13.5 (4.4)	14.4 (5.7)	13.9 (4.9)
Pain intensity NRS^f^, mean (SD)	6.3 (1.6)	7.1 (1.8)	6.7 (1.7)
NPR^g^, mean (SD)	13.6 (10.0)	15.7 (9.8)	14.5 (9.9)
PDI^h^, mean (SD)	39.9 (11.6)	41.3 (10.3)	40.5 (10.9)
EQ5-VAS^i^, mean (SD)	43.9 (17.1)	43.3 (17.7)	43.7 (17.2)
Duration of pain problems >5 years, n (%)	20 (67)	14 (58)	34 (63)
Duration of sleep problems >5 years, n (%)	23 (77)	19 (79)	42 (78)
The pain problem preceded the sleep problem, n (%)	18 (60)	14 (58)	32 (59)

^a^ICBT-i: internet-based cognitive behavioral therapy for insomnia.

^b^AR: applied relaxation.

^c^ISI: Insomnia Severity Index.

^d^GAD-7: Generalized Anxiety Disorder–7 items.

^e^PHQ-9: Patient Health Questionnaire–9 items.

^f^NRS: Numeric Rating Scale.

^g^NPR: number of pain regions.

^h^PDI: Pain Disability Index.

^i^EQ5-VAS: European Quality of Life 5-Dimension Visual Analog Scale.

### Dropout Analysis

Comparisons between dropouts and completers revealed that participants who dropped out were more likely to be older, had lower education and pain disability, and had higher levels of depression and anxiety (Table S4 in Multimedia Appendix 2).

### Primary Outcome

**Figure 2:**
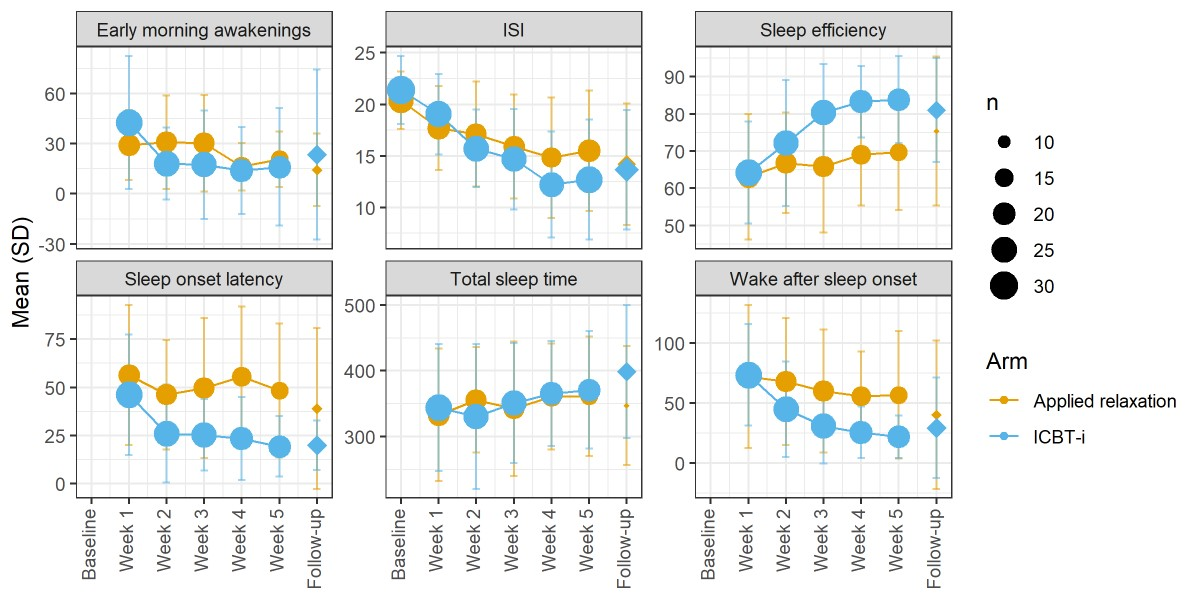
Sleep measures for internet-based cognitive behavioral therapy for insomnia (ICBT-i) and applied relaxation over time. Baseline data are presented only for Insomnia Severity Index (ISI). The active treatment period is illustrated by dots, and follow-up data are illustrated by diamonds. Note that y-axes are not illustrated from 0.

**Figure 3:**
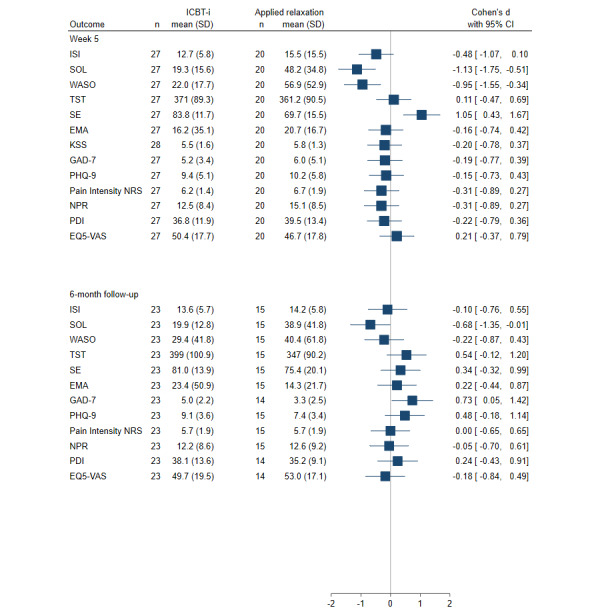
Effect sizes (random effects) for internet-based cognitive behavioral therapy for insomnia (ICBT-i) and applied relaxation at week 5 and 6-month follow-up. EMA: early morning awakenings; EQ5-VAS: European Quality of Life 5-Dimension Visual Analog Scale; GAD-7: Generalized Anxiety Disorder–7 items; ISI: Insomnia Severity Index; KSS: Karolinska Sleepiness Scale; NPR: number of pain regions; NRS: Numeric Rating Scale; PDI: Pain Disability Index; PHQ-9: Patient Health Questionnaire–9 items; SE: sleep efficiency; SOL: sleep onset latency; TST: total sleep time; WASO: wake time after sleep onset.

At the posttreatment phase, the mean improvement in the ICBT-I group was 8.4 (SD 4.7), and the mean improvement in the applied relaxation group was 5.0 (SD 5.4). The mixed model showed a significant immediate interaction effect (time by treatment) on the ISI, such that the ICBT-i group showed a greater rate of improvement (Figure 2 and Table 2). At follow-up for the ICBT-I group, the mean change score was 6.7 (SD 5.4); for the control group, the mean change score was 6.1 (SD 5.2). At the 6-month follow-up, there was no difference between treatments according to the ISI (Table S5 in Multimedia Appendix 2). Figure 3 shows the between-group effect sizes based on random effects (Cohen *d*) for all outcomes at week 5-and follow-up.

**Table 2:** Mixed models for ISI^a^ from baseline to week 5 and for weekly sleep diary and KSS^b^ scores from the beginning of the treatment (week 1) to the end of the treatment (week 5).

			Estimate (SE; 95% CI)		*P* value
**ISI**					
	Intercept (AIC^c^ 1414.2)	19.66 (0.70; 18.2 to 21.0)		*<.001* ^d^	
	Treatment	1.14 (0.94; −0.69 to 2.98)		.22	
	Time (week 5)	−1.02 (0.23; −1.47 to −0.55)		*<.001*	
	Time (week 5)×treatment	−0.72 (0.31; −1.33 to −0.11)		*.02*	
**Sleep onset latency**					
	Intercept (AIC 2022.4)	53.03 (7.37; 38.5 to 67.4)		*<.001*	
	Treatment	−9.52 (9.62; −28.1 to 9.60)		.34	
	Time (week 5)	0.68 (5.00; −9.13 to 10.4)		.89	
	TimeQ^e^ (week 5)	−0.58 (0.88; −2.32 to 1.14)		.50	
	Time (week 5)×treatment	−13.77 (6.55; −26.5 to −0.09)		*.04*	
	TimeQ (week 5)×treatment	2.38 (1.15; 0.13 to 4.63)		*.04*	
**Wake time after sleep onset**					
	Intercept (AIC 1957.3)	73.24 (17.59; 38.7 to 107.7)		*<.001*	
	Treatment	−2.27 (18.77; −39.0 to 34.5)		.90	
	Time (week 5)	−5.23 (4.57; −14.4 to 3.43)		.24	
	TimeQ (week 5)	0.29 (0.41; −0.50 to 1.09)		.47	
	Time (week 5)×treatment	−20.98 (5.96; −32.6 to −9.30)		*<.001*	
	TimeQ (week 5)×treatment	4.45 (0.53; 0.53 to 3.41)		*<.001*	
**Total sleep time**					
	Intercept (AIC 2239.3)	336.75 (20.93; 295.7 to 377.7)		*<.001*	
	Treatment	1.37 (27.30; −52.1 to 54.9)		.96	
	Time (week 5)	5.77 (3.57; −1.22 to 12.7)		.11	
	Time (week 5)×treatment	−0.92 (4.67; −10.1 to 8.23)		.84	
**Sleep efficiency**					
	Intercept (AIC 1500.6)	63.49 (3.71; 56.2 to 70.7)		*<.001*	
	Treatment	0.79 (4.46; −7.96 to 9.55)		.86	
	Time (week 5)	1.34 (0.90; −0.43 to 3.12)		.14	
	TimeQ (week 5)	0.03 (0.16; −0.27 to 0.35)		.81	
	Time (week 5)×treatment	8.29 (0.18; 5.97 to 10.6)		*<.001*	
	TimeQ (week 5)×treatment	−1.44 (0.20; −1.85 to −1.03)		*<.001*	
**Early morning awakenings**					
	Intercept (AIC 1834.3)	30.40 (6.18; 18.3 to 42.5)		*<.001*	
	Treatment	9.80 (8.53; −6.91 to 26.5)		.26	
	Time (week 5)	0.36 (2.16; −3.87 to 4.58)		.87	
	TimeQ (week 5)	−0.93 (0.35; −1.63 to −0.25)		*.008*	
	Time (week 5)×treatment	−20.65 (2.82; −26.1 to −15.2)		*<.001*	
	TimeQ (week 5)×treatment	4.50 (0.46; 3.60 to 5.41)		*<.001*	
**KSS**					
	Intercept (AIC 585.8)	6.15 (0.21; 5.73 to 6.56)		*<.001*	
	Treatment	−0.07 (0.28; −0.49 to 0.62)		.82	
	Time (week 5)	−0.18 (0.06; −0.29 to −0.07)		*.002*	
	Time (week 5)×treatment	0.07 (0.07; −0.07 to 0.21)		.33	

^a^ISI: Insomnia Severity Index.

^b^KSS: Karolinska Sleepiness Scale.

^c^AIC: Akaike information criterion.

^d^Italics indicates statistically significant results.

^e^TimeQ: time quadratic.

### Sleep Parameters

The mean values for sleep parameters at the posttreatment and follow-up periods are shown in Figure 2. The mixed model shows a significant time (quadratic)-by-treatment immediate effect on SOL, WASO, sleep efficiency, and EMA during the active treatment phase. Compared with the applied relaxation group, the ICBT-I group showed a more rapid increase in sleep efficiency and a more rapid decrease in EMA, SOL, and WASO (Table 2). At the 6-month follow-up, there was no statistically significant time-by-treatment effects on sleep diary measures when n dropped to 5 in the control group (Table S5 in Multimedia Appendix 2). Figure 3 shows the between-group effect sizes based on random effects (Cohen *d*) for all outcomes at week 5-and follow-up.

### Daytime Sleepiness

The mean KSS values are shown in Figure S1 in Multimedia Appendix 2. The mixed models revealed either no treatment or time-by-treatment immediate effect on KSS (Table 2). That is, both treatment arms changed over time (active treatment phase), but there was no significant difference in the slope at any time point. Figure 3 shows the between-group effect sizes based on random effects (Cohen *d*) for all outcomes at week 5-and follow-up.

### Other Secondary Outcomes

The mean values and their SDs at week 5-and follow-up are presented in Table S6 in Multimedia Appendix 2. No significant differences based on mixed models were found for most of the secondary outcomes with 2-time measurements (before vs after the treatment; Table 3). At follow-up, the mixed models showed significant time effects for PHQ-9 (depression) and GAD-7 (anxiety) versus those at week 5 (Table S7 in Multimedia Appendix 2). Figure 3 shows the between-group effect sizes based on random effects (Cohen *d*) for all outcomes at week 5 and follow-up.

**Table 3:** Mixed models for secondary outcomes from baseline to week 5.

Outcomes			Estimate (SE; 95% CI)		*P* value
**GAD-7^a^**					
	Intercept (AIC^b^ 591.2)	8.25 (0.94; 6.42 to 10.1)		*<.001* ^c^	
	Treatment	−0.81 (1.25; −3.28 to 1.64)		.52	
	Time (week 5)	−1.81 (1.05; −3.87 to 0.24)		.09	
	Time (week 5)×treatment	−0.16 (1.38; −2.88 to 2.55)		.90	
**PHQ-9^d^**					
	Intercept (AIC 618.0)	14.17 (1.07; 12.3 to 16.5)		*<.001*	
	Treatment	−0.88 (1.44; −3.71 to 1.94)		.54	
	Time (week 5)	−3.92 (1.18; −6.23 to −1.61)		*.002*	
	Time (week 5)×treatment	0.15 (1.56; −2.91 to 3.22)		.92	
**Pain Intensity NRS^e^**					
	Intercept (AIC 369.4)	7.16 (0.34; 6.50 to 7.83)		*<.001*	
	Treatment	−0.87 (0.46; −1.76 to 0.03)		.06	
	Time (week 5)	−0.42 (0.30; −1.00 to 0.16)		.16	
	Time (week 5)×treatment	0.25 (0.39; −0.48 to 1.06)		.47	
**NPR^f^**					
	Intercept (AIC 670.7)	15.71 (1.92; 11.9 to 19.5)		*<.001*	
	Treatment	−2.08 (2.58; −7.14 to 2.99)		.43	
	Time (week 5)	−1.49 (0.95; −3.35 to 0.36)		.12	
	Time (week 5)×treatment	1.51 (1.25; −0.93 to 3.96)		.23	
**PDI^g^**					
	Intercept (AIC 764.6)	41.30 (2.42; 36.5 to 46.1)		*<.001*	
	Treatment	−1.43 (3.24; −7.79 to 4.93)		.66	
	Time (week 5)	−2.44 (2.09; −6.53 to 1.65)		.25	
	Time (week 5)×treatment	−0.85 (2.76; −6.26 to 4.56)		.76	
**EQ5-VAS^h^**					
	Intercept (AIC 856.1)	43.33 (3.60; 36.3 to 50.4)		*<.001*	
	Treatment	0.63 (4.84; −8.84 to 10.1)		.89	
	Time (week 5)	3.13 (3.60; −3.93 to 10.2)		.39	
	Time (week 5)×Treatment	3.32 (4.77; −6.02 to 12.7)		.49	

^a^GAD-7: Generalized Anxiety Disorder–7 items.

^b^AIC: Akaike information criterion.

^c^Italics indicates statistically significant results.

^d^PHQ-9: Patient Health Questionnaire–9 items.

^e^NRS: Numeric Rating Scale.

^f^NPR: number of pain regions.

^g^PDI: Pain Disability Index.

^h^EQ5-VAS: European Quality of Life 5-Dimension Visual Analog Scale.

### Treatment Credibility

Treatment credibility measures are presented in Table S8 in Multimedia Appendix 2. There were no significant group differences at week 2 (end of week 2 of treatment), week 5 (after the treatment), or follow-up.

### Compliance

The median for completed modules was 4.5 (IQR 3-5) for the ICBT-i group and 4 (IQR 1.75-5) for the control group. The difference between the modules was not statistically significant (*P*=.46).

### Negative Effects

The most frequently reported negative effect in both the treatment arms was increased stress. In the ICBT-i group, the second most reported negative effect was the experience that the treatment did not suit the patient. In contrast, the second most reported negative effect in the applied relaxation control condition was that participants stopped believing that there was available help. Table S9 in Multimedia Appendix 2 provides a comprehensive description of the negative effects.

## Discussion

### Principal Findings

In this study, we investigated the effects of ICBT-ion insomnia severity and other sleep and pain-related parameters in patients with chronic pain. Our results suggest that ICBT-i decreases insomnia severity and related nighttime symptoms. In particular, we found that the ICBT-i group, compared with the applied relaxation group, had greater immediate improvements in insomnia symptoms as measured by ISI, WASO, sleep efficiency, and EMA. The immediate effect (within-subject) of ICBT-i on ISI was clinically significant according to definition by Morin et al [5]. After 6 months, the group means approached each other, and no differences were confirmed on ISI (or other sleep parameters). Overall, our findings are consistent with previous studies suggesting that CBT-i delivers posttreatment improvements in insomnia and sleep symptoms, irrespective of the format [61-65]. However, further research is needed to investigate the effect of ICBT-i on a larger sample size of people with chronic pain.

### Strengths and Limitations

The strengths of this study are its randomized design with an active control condition, low posttreatment attrition rate (13%), and clinical context. However, the attrition rate at follow-up was higher (30%). However, this study has several limitations. First, the small sample size makes it underpowered to detect small changes in pain and other symptoms and to adjust for confounders or to perform subgroup analyses, for example, by sex or educational level. The response to study invitations was surprisingly weak despite the invitations being directed to current and former patients identified with moderate to severe insomnia symptoms. This weak response might have been due to the relatively extensive treatments and commitment behavior changes require. Therefore, our results should be interpreted with caution owing to the underpowered nature of the study. This study also lacked objective sleep measures, such as accelerometer-based biosensors, a method that was excluded because of the priority to keep the treatment and assessment as brief as possible. The inclusion of these biosensors would also have made participation more complicated, with an increased risk for nonparticipation or dropout. Furthermore, our sample consisted primarily of women (45/54, 83%); therefore, a selection bias should not be ignored, regardless of the randomization procedure. This sex skew might be due to fact that insomnia symptoms are more common in women (aged >45 years) [66] and that the source of recruitment consisted of approximately 68.3% (123/180) women [4]. However, our results may still be considered ecologically valid. In addition, this study stands out with respect to the treatment length. Unpublished data from a previous treatment study by members of our group showed that SOL was the shortest after 5 weeks of treatment; therefore, 5 weeks/modules were chosen in this study. This study continued for another 2 weeks, but because an aim of this study is to develop a brief ICBT-i intervention, we decided to end the active treatment phase after 5 weeks. It can be argued that this was premature and that the treatment effect was limited by this decision. It is also possible that the effects of ICBT-i would have been better consolidated and maintained if treatment, sleep monitoring, and therapist support had lasted longer. Despite lower baseline ISI values, Jungquist et al [67] achieved a larger decrease in absolute number (13 points) on the same outcome measure after their 8-week CBT-i treatment. It may also be argued that the design of the active control condition, with a specific treatment factor (ie, applied relaxation), decreased the contrast with the experimental condition [68].

On the basis of these data, it is impossible to rule out whether the lack of time-by-treatment effect at follow-up is a result of a power issue or whether the 2 treatment conditions are equally effective in reducing insomnia symptoms in the long term. Given that 78% (42/54) of the sample had a sleeping problem duration of >5 years, explanations such as regression to the mean or spontaneous recovery are less likely. It cannot be ruled out that some overlap exists between treatment arms; therefore, the observed improvements, at least in part, may be due to common factors between the 2 arms. Both arms had access to the responsive sleep diary, calculating total sleep time, time in bed, SOL, WASO, EMA, and sleep efficiency. This could lead to unintentional behavior change in the control condition through the participants’ own inferences of aggregated sleep data, although the addition of a wait-list control could have offered a valuable reference category. In contrast, a wait-list would have increased the power problem even more, given that further recruitment would not be possible. No measures of therapist fidelity were collected, but the standardized treatment content in both treatments, along with the supervision of therapists, helped maintain treatment fidelity [69]. However, differences in therapist support may exist, although the same therapists provided both treatments. Moreover, the participants in the control condition completed fewer treatment modules, which may constitute a major limitation. In addition, because blinding is problematic in this research field, it is also plausible to assume that there is a substantial risk of contamination between both treatment arms. Finally, the results from the 6-month follow-up were rather uncertain, as the response rate declined considerably. This decline is especially true for sleep parameters based on the sleep diary data.

In addition, there are some general limitations related to internet treatments. [70]. Although a therapist alliance seems possible to establish, the ability to monitor the patients’ progress and setbacks seems to be limited by this format. Consequently, the ability to make adaptations is limited, particularly when the treatment content is predefined. This study offers therapist support that likely contributes to increased adherence, which has been reported to be poor in previous studies. The exclusion of non-Swedish speakers and people lacking internet connections is another limitation related to this format.

### Comparison With Previous Work

We found that immediate effects on ISI were larger in ICBT-i, but over the follow-up period, initial gains seemed to decline, but the applied control condition continued to improve. This finding was not unexpected, as results from a meta-analysis also illustrated that the long-term effects of CBT on insomnia are unclear [71]. Moreover, both treatments may be effective, but through different mechanisms and in different time perspectives. Applied relaxation targets arousal, which could hinder or interrupt sleep [38], whereas ICBT-i targets increasing the sleep homeostatic sleep load and establishing a sound circadian rhythm [35,36,72]. Therefore, we can speculate that a combination of both these treatments, which is common in CBT-i, would lead to an even better outcome. Notably, despite our intention to control for treatment extent, the applied relaxation group as a comparison arm completed a slightly less treatment content than the ICBT-i group. Furthermore, immediate time-by-treatment effects were found for the sleep parameters WASO, sleep efficiency, and EMA. SOL reached significance only at 1 time point. Changes from baseline are comparable with those of other studies, except for EMA, which previous studies failed to show any improvement or did not report [67]. The total absence of long-term time-by-treatment effects is likely, which could be explained by this study’s inability to collect sleep diary data at follow-up, especially from participants in the control condition.

This study also highlighted that both web-based treatments improved psychological outcomes (depression and anxiety) over time, making these findings difficult to interpret. One may argue that both conditions improved these symptoms during the follow-up period, but unfortunately, the design of this study did not include any measure of the extent to which participants continued to apply the methods taught throughout treatment. It is also possible that medication titration might have affected these outcomes, as the medication was not specifically addressed in both conditions. Of the 30 participants in the experimental condition and 24 in the control condition, 29 (97%) and 23 (96%) at baseline reported taking prescription medication for their symptoms (including pain killers, benzodiazepine receptor agonists, or antidepressants), respectively. In addition, no exploratory analysis could be applied because changes in medication after the follow-up period were not monitored.

In contrast, none of the treatments had a significant effect on the number of pain regions or pain intensity at the posttreatment or follow-up period. Despite high initial values and clear immediate effect on insomnia severity (at least in ICBT-i), no time or time-by-treatment, effects on pain measures were found. These findings are supported by previous research in this field, which suggests that the effects of CBT-ion pain-related outcomes are highly unreliable [73]. The nonsignificant effects on pain measures may also be due to the strong correlation between insomnia and pain intensity. If the correlation is strong, a treatment effect on pain intensity is more likely than if a weak (but significant) correlation has been observed between the 2 variables [55,56]. In addition, two meta-analyses by Tang et al [17] and Selvanathan et al [74] also found overall small effects on pain intensity. Again, the lack of results in this study could be a consequence of the small sample size and low power. An alternative interpretation is that insomnia symptoms in participants with long-standing conditions can be improved without necessarily affecting pain intensity.

Finally, the most reported negative effect in both treatment arms (item 2: I felt like I was under more stress) was also the most frequent in several other CBT studies on different diagnoses [58]. The second most reported negative outcome in the active control condition (item 19: I stopped believing that there was available help) was excluded in the revised version of the NEQ because of poor goodness of fit [58]. It is also possible that this item expresses the absence of an expected treatment effect in nonresponders, rather than a negative effect of treatment. Interestingly, 22% (7/30) of the participants in the ICBT-i group and 17% (4/24) of the controls reported more problems with sleep (item 1). The item does not indicate whether this refers to long- or short-term problems; however, the impact of these negative effects seems to be limited to the means presented here.

### Conclusion and Clinical Implications

Compared with applied relaxation, ICBT-i led to a greater decline in insomnia symptoms. Treatment gains were largely preserved over 6 months, but the group difference decreased as the control condition continued to improve over this period. Furthermore, applied relaxation was more successful in reducing comorbid anxiety and depressive symptoms in the long term. Further studies are needed to examine whether a combination of ICBT-i and applied relaxation leads to even larger reductions in insomnia symptoms or what necessary components of ICBT-i work best for insomnia in patients with chronic pain. Nevertheless, it was possible to achieve clinically significant improvement in insomnia symptoms via this brief 5-week internet treatment. If our finding that a brief CBT-i intervention (possibly in combination with applied relaxation) affects insomnia in patients with chronic pain can be confirmed in larger studies, this intervention may be practically applicable in the clinical setting. This could be an important advance in the treatment arsenal, as a large proportion of patients with chronic pain also experience insomnia.

## Appendix

Multimedia Appendix 1 CONSORT-eHEALTH checklist (V 1.6.1).

Multimedia Appendix 2 Supplementary information on methods and results.

## Abbreviations

CBT: cognitive behavioral therapy
CBT-i: cognitive behavioral therapy for insomnia
CONSORT: Consolidated Standards of Reporting Trials
GAD-7: Generalized Anxiety Disorder–7 items
ICBT-i: internet-based cognitive behavioral therapy for insomnia
ISI: Insomnia Severity Index
KSS: Karolinska Sleepiness Scale
NEQ: Negative Effects Questionnaire
PDI: Pain Disability Index
PHQ-9: Patient Health Questionnaire–9 items
RCT: randomized controlled trial
SOL: sleep onset latency
WASO: wake time after sleep onset
