# Supplementary material for: Internet-Delivered Cognitive Behavioral Therapy for Insomnia Comorbid With Chronic Pain: Randomized Controlled Trial
Source: J Med Internet Res. 2022 Apr 29;24(4):e29258. doi: 10.2196/29258 (PMC9107050; doi:10.2196/29258)
Supplement: Multimedia Appendix 1 [file jmir_v24i4e29258_app1.pdf]

CONSORT-EHEALTH (V 1.6.1) - Submission/Publication Form

The CONSORT-EHEALTH checklist is intended for authors of randomized trials evaluating web-based and Internet-based applications/interventions, including mobile interventions, electronic games (incl multiplayer games), social media, certain telehealth applications, and other interactive and/or networked electronic applications. Some of the items (e.g. all subitems under item 5 - description of the intervention) may also be applicable for other study designs.

The goal of the CONSORT EHEALTH checklist and guideline is to be
a) a guide for reporting for authors of RCTs,
b) to form a basis for appraisal of an ehealth trial (in terms of validity)

CONSORT-EHEALTH items/subitems are MANDATORY reporting items for studies published in the Journal of Medical Internet Research and other journals / scientific societies endorsing the checklist.

Items numbered 1., 2., 3., 4a., 4b etc are original CONSORT or CONSORT-NPT (non-pharmacologic treatment) items.
Items with Roman numerals (i., ii, iii, iv etc.) are CONSORT-EHEALTH extensions/clarifications.

As the CONSORT-EHEALTH checklist is still considered in a formative stage, we would ask that you also RATE ON A SCALE OF 1-5 how important/useful you feel each item is FOR THE PURPOSE OF THE CHECKLIST and reporting guideline (optional).

Mandatory reporting items are marked with a red *.
In the textboxes, either copy & paste the relevant sections from your manuscript into this form - please include any quotes from your manuscript in QUOTATION MARKS,
or answer directly by providing additional information not in the manuscript, or elaborating on why the item was not relevant for this study.

YOUR ANSWERS WILL BE PUBLISHED AS A SUPPLEMENTARY FILE TO YOUR PUBLICATION IN JMIR AND ARE CONSIDERED PART OF YOUR PUBLICATION (IF ACCEPTED).

Please fill in these questions diligently. Information will not be copyedited, so please use proper spelling and grammar, use correct capitalization, and avoid abbreviations.

DO NOT FORGET TO SAVE AS PDF _AND_ CLICK THE SUBMIT BUTTON SO YOUR ANSWERS ARE IN OUR DATABASE !!!

Citation Suggestion (if you append the pdf as Appendix we suggest to cite this paper in the caption):
Eysenbach G, CONSORT-EHEALTH Group
CONSORT-EHEALTH: Improving and Standardizing Evaluation Reports of Web-based and Mobile Health Interventions
J Med Internet Res 2011;13(4):e126
URL: http://www.jmir.org/2011/4/e126/
doi: 10.2196/jmir.1923
PMID: 22209829

consortonly@gmail.com (Delas inte) Byt konto

Utkastet har återställts

*Obligatorisk

Your name *

First Last

Tobias Wiklund

Primary Affiliation (short), City, Country *

University of Toronto, Toronto, Canada

Linköping University, Linköping, Sweden

Your e-mail address *

abc@gmail.com

wiklund86@gmail.com

Title of your manuscript *

Provide the (draft) title of your manuscript.

Internet-delivered cognitive behavioural therapy for insomnia comorbid with chronic pain: A randomised controlled trial

Name of your App/Software/Intervention *

If there is a short and a long/alternate name, write the short name first and add the long name in brackets.

SoV-studien (Sömn och Värk-studien)

Evaluated Version (if any)

e.g. "V1", "Release 2017-03-01", "Version 2.0.27913"

Ditt svar

Language(s) *

What language is the intervention/app in? If multiple languages are available, separate by comma (e.g. "English, French")

Swedish

URL of your Intervention Website or App

e.g. a direct link to the mobile app on app in appstore (itunes, Google Play), or URL of the website. If the intervention is a DVD or hardware, you can also link to an Amazon page.

https://www.iterapi.se/sites/sovstudien/

URL of an image/screenshot (optional)

Ditt svar

Accessibility *

Can an enduser access the intervention presently?

- ○ access is free and open
- ○ access only for special usergroups, not open
- ○ access is open to everyone, but requires payment/subscription/in-app purchases
- ◉ app/intervention no longer accessible
- ○ Övrigt:

Primary Medical Indication/Disease/Condition *

e.g. "Stress", "Diabetes", or define the target group in brackets after the condition, e.g. "Autism (Parents of children with)", "Alzheimers (Informal Caregivers of)"

Insomnia secondary to chronic pain

Primary Outcomes measured in trial *

comma-separated list of primary outcomes reported in the trial

Insomnia Severity Index

Secondary/other outcomes

Are there any other outcomes the intervention is expected to affect?

Sleep efficiency, sleep onset latency, wake time after sleep onset, early morning awakenings, karolinska sleepiness scale, PHQ-9, GAD-7

Recommended "Dose" *

What do the instructions for users say on how often the app should be used?

- ○ Approximately Daily
- ◉ Approximately Weekly
- ○ Approximately Monthly
- ○ Approximately Yearly
- ○ "as needed"
- ○ Övrigt:

Approx. Percentage of Users (starters) still using the app as recommended after 3 months *

- (selected) unknown / not evaluated
- 0-10%
- 11-20%
- 21-30%
- 31-40%
- 41-50%
- 51-60%
- 61-70%
- 71%-80%
- 81-90%
- 91-100%
- Övrigt:

Overall, was the app/intervention effective? *

- ( ) yes: all primary outcomes were significantly better in intervention group vs control
- (•) partly: SOME primary outcomes were significantly better in intervention group vs control
- ( ) no statistically significant difference between control and intervention
- ( ) potentially harmful: control was significantly better than intervention in one or more outcomes
- ( ) inconclusive: more research is needed
- ( ) Övrigt:

Article Preparation Status/Stage *

At which stage in your article preparation are you currently (at the time you fill in this form)

- ( ) not submitted yet - in early draft status
- ( ) not submitted yet - in late draft status, just before submission
- ( ) submitted to a journal but not reviewed yet
- ( ) submitted to a journal and after receiving initial reviewer comments
- (•) submitted to a journal and accepted, but not published yet
- ( ) published
- ( ) Övrigt:

Journal *

If you already know where you will submit this paper (or if it is already submitted), please provide the journal name (if it is not JMIR, provide the journal name under "other")

- ○ not submitted yet / unclear where I will submit this
- ◉ Journal of Medical Internet Research (JMIR)
- ○ JMIR mHealth and UHealth
- ○ JMIR Serious Games
- ○ JMIR Mental Health
- ○ JMIR Public Health
- ○ JMIR Formative Research
- ○ Other JMIR sister journal
- ○ Övrigt:

Is this a full powered effectiveness trial or a pilot/feasibility trial? *

- ◉ Pilot/feasibility
- ○ Fully powered

Manuscript tracking number *

If this is a JMIR submission, please provide the manuscript tracking number under "other" (The ms tracking number can be found in the submission acknowledgement email, or when you login as author in JMIR. If the paper is already published in JMIR, then the ms tracking number is the four-digit number at the end of the DOI, to be found at the bottom of each published article in JMIR)

- ○ no ms number (yet) / not (yet) submitted to / published in JMIR
- ◉ Övrigt: 29258

TITLE AND ABSTRACT

1a) TITLE: Identification as a randomized trial in the title

1a) Does your paper address CONSORT item 1a? *

I.e does the title contain the phrase "Randomized Controlled Trial"? (if not, explain the reason under "other")

- ◉ yes
- ○ Övrigt:

1a-i) Identify the mode of delivery in the title

Identify the mode of delivery. Preferably use “web-based” and/or “mobile” and/or “electronic game” in the title. Avoid ambiguous terms like “online”, “virtual”, “interactive”. Use “Internet-based” only if Intervention includes non-web-based Internet components (e.g. email), use “computer-based” or “electronic” only if offline products are used. Use “virtual” only in the context of “virtual reality” (3-D worlds). Use “online” only in the context of “online support groups”. Complement or substitute product names with broader terms for the class of products (such as “mobile” or “smart phone” instead of “iphone”), especially if the application runs on different platforms.

	1	2	3	4	5	
subitem not at all important	○	○	○	◉	○	essential

Rensa markering

Does your paper address subitem 1a-i? *

Copy and paste relevant sections from manuscript title (include quotes in quotation marks "like this" to indicate direct quotes from your manuscript), or elaborate on this item by providing additional information not in the ms, or briefly explain why the item is not applicable/relevant for your study

"Internet-delivered cognitive behavioural therapy "

1a-ii) Non-web-based components or important co-interventions in title

Mention non-web-based components or important co-interventions in title, if any (e.g., “with telephone support”).

	1	2	3	4	5	
subitem not at all important	(●)	( )	( )	( )	( )	essential

Rensa markering

Does your paper address subitem 1a-ii?

Copy and paste relevant sections from manuscript title (include quotes in quotation marks "like this" to indicate direct quotes from your manuscript), or elaborate on this item by providing additional information not in the ms, or briefly explain why the item is not applicable/relevant for your study

Ditt svar

1a-iii) Primary condition or target group in the title

Mention primary condition or target group in the title, if any (e.g., “for children with Type I Diabetes”)
Example: A Web-based and Mobile Intervention with Telephone Support for Children with Type I Diabetes: Randomized Controlled Trial

	1	2	3	4	5	
subitem not at all important	( )	( )	( )	(●)	( )	essential

Rensa markering

Does your paper address subitem 1a-iii? *

Copy and paste relevant sections from manuscript title (include quotes in quotation marks "like this" to indicate direct quotes from your manuscript), or elaborate on this item by providing additional information not in the ms, or briefly explain why the item is not applicable/relevant for your study

"insomnia comorbid with chronic pain"

1b) ABSTRACT: Structured summary of trial design, methods, results, and conclusions

NPT extension: Description of experimental treatment, comparator, care providers, centers, and blinding status.

1b-i) Key features/functionalities/components of the intervention and comparator in the METHODS section of the ABSTRACT

Mention key features/functionalities/components of the intervention and comparator in the abstract. If possible, also mention theories and principles used for designing the site. Keep in mind the needs of systematic reviewers and indexers by including important synonyms. (Note: Only report in the abstract what the main paper is reporting. If this information is missing from the main body of text, consider adding it)

	1	2	3	4	5	
subitem not at all important	○	○	●	○	○	essential

Rensa markering

Does your paper address subitem 1b-i? *

Copy and paste relevant sections from the manuscript abstract (include quotes in quotation marks "like this" to indicate direct quotes from your manuscript), or elaborate on this item by providing additional information not in the ms, or briefly explain why the item is not applicable/relevant for your study

"internet-based cognitive behavioral therapy for insomnia"; "applied relaxation"

1b-ii) Level of human involvement in the METHODS section of the ABSTRACT

Clarify the level of human involvement in the abstract, e.g., use phrases like "fully automated" vs. "therapist/nurse/care provider/physician-assisted" (mention number and expertise of providers involved, if any). (Note: Only report in the abstract what the main paper is reporting. If this information is missing from the main body of text, consider adding it)

	1	2	3	4	5	
subitem not at all important	○	○	●	○	○	essential

Rensa markering

Does your paper address subitem 1b-ii?

Copy and paste relevant sections from the manuscript abstract (include quotes in quotation marks "like this" to indicate direct quotes from your manuscript), or elaborate on this item by providing additional information not in the ms, or briefly explain why the item is not applicable/relevant for your study

No, not in the abstract but in the paper

1b-iii) Open vs. closed, web-based (self-assessment) vs. face-to-face assessments in the METHODS section of the ABSTRACT

Mention how participants were recruited (online vs. offline), e.g., from an open access website or from a clinic or a closed online user group (closed usergroup trial), and clarify if this was a purely web-based trial, or there were face-to-face components (as part of the intervention or for assessment). Clearly say if outcomes were self-assessed through questionnaires (as common in web-based trials). Note: In traditional offline trials, an open trial (open-label trial) is a type of clinical trial in which both the researchers and participants know which treatment is being administered. To avoid confusion, use “blinded” or “unblinded” to indicated the level of blinding instead of “open”, as “open” in web-based trials usually refers to “open access” (i.e. participants can self-enrol). (Note: Only report in the abstract what the main paper is reporting. If this information is missing from the main body of text, consider adding it)

	1	2	3	4	5	
subitem not at all important	○	○	◉	○	○	essential

Rensa markering

Does your paper address subitem 1b-iii?

Copy and paste relevant sections from the manuscript abstract (include quotes in quotation marks "like this" to indicate direct quotes from your manuscript), or elaborate on this item by providing additional information not in the ms, or briefly explain why the item is not applicable/relevant for your study

"Participants were selected via the Swedish Quality Registry for Pain Rehabilitation"; "Participants with an ISI score of >14 (moderate to severe insomnia symptoms) and aged 18-65 years were asked to participate in the trial either by their physician or via postal invitation. Retrospective analyses were performed to identify former patients with an ISI score of >14 over the previous 2 years. If interested, participants registered on the website and completed another survey"

1b-iv) RESULTS section in abstract must contain use data

Report number of participants enrolled/assessed in each group, the use/uptake of the intervention (e.g., attrition/adherence metrics, use over time, number of logins etc.), in addition to primary/secondary outcomes. (Note: Only report in the abstract what the main paper is reporting. If this information is missing from the main body of text, consider adding it)

	1	2	3	4	5	
subitem not at all important	○	○	◉	○	○	essential

Rensa markering

Does your paper address subitem 1b-iv?

Copy and paste relevant sections from the manuscript abstract (include quotes in quotation marks "like this" to indicate direct quotes from your manuscript), or elaborate on this item by providing additional information not in the ms, or briefly explain why the item is not applicable/relevant for your study

"We included 54 patients (mean age 49.3, SD 12.3 years) who were randomly assigned to the ICBT-i condition and
24 to an active control condition (applied relaxation).", but no report on attrition

1b-v) CONCLUSIONS/DISCUSSION in abstract for negative trials

Conclusions/Discussions in abstract for negative trials: Discuss the primary outcome - if the trial is negative (primary outcome not changed), and the intervention was not used, discuss whether negative results are attributable to lack of uptake and discuss reasons. (Note: Only report in the abstract what the main paper is reporting. If this information is missing from the main body of text, consider adding it)

	1	2	3	4	5	
subitem not at all important	◉	○	○	○	○	essential

Rensa markering

Does your paper address subitem 1b-v?

Copy and paste relevant sections from the manuscript abstract (include quotes in quotation marks "like this" to indicate direct quotes from your manuscript), or elaborate on this item by providing additional information not in the ms, or briefly explain why the item is not applicable/relevant for your study

Ditt svar

INTRODUCTION

2a) In INTRODUCTION: Scientific background and explanation of rationale

2a-i) Problem and the type of system/solution

Describe the problem and the type of system/solution that is object of the study: intended as stand-alone intervention vs. incorporated in broader health care program? Intended for a particular patient population? Goals of the intervention, e.g., being more cost-effective to other interventions, replace or complement other solutions? (Note: Details about the intervention are provided in “Methods” under 5)

	1	2	3	4	5	
subitem not at all important	○	○	○	◉	○	essential

Rensa markering

Does your paper address subitem 2a-i? *

Copy and paste relevant sections from the manuscript (include quotes in quotation marks "like this" to indicate direct quotes from your manuscript), or elaborate on this item by providing additional information not in the ms, or briefly explain why the item is not applicable/relevant for your study

"Open-source
and evidence-based treatments will improve care by addressing
a common aspect of chronic pain, that is, insomnia symptoms.
In addition, the implementation of internet-based cognitive
behavioral therapy for insomnia (ICBT-i) has immense potential
in terms of cost-effectiveness, as the time per patient is shorter
than face-to-face CBT-i [25,26]. Previous work from members
of our group on persons with insomnia disorder showed that
CBT-i provided via the internet (ICBT-i) has comparable
outcomes to cognitive behavioral therapy (CBT) provided in a
group setting"

2a-ii) Scientific background, rationale: What is known about the (type of) system

Scientific background, rationale: What is known about the (type of) system that is the object of the study (be sure to discuss the use of similar systems for other conditions/diagnoses, if appropiate), motivation for the study, i.e. what are the reasons for and what is the context for this specific study, from which stakeholder viewpoint is the study performed, potential impact of findings [2]. Briefly justify the choice of the comparator.

	1	2	3	4	5	
subitem not at all important	○	○	○	●	○	essential

Rensa markering

Does your paper address subitem 2a-ii? *

Copy and paste relevant sections from the manuscript (include quotes in quotation marks "like this" to indicate direct quotes from your manuscript), or elaborate on this item by providing additional information not in the ms, or briefly explain why the item is not applicable/relevant for your study

"Patients with chronic pain often experience insomnia"; "However, there is no available data on ICBT-i
targeting insomnia symptoms in patients with chronic pain.";

2b) In INTRODUCTION: Specific objectives or hypotheses

Does your paper address CONSORT subitem 2b? *

Copy and paste relevant sections from the manuscript (include quotes in quotation marks "like this" to indicate direct quotes from your manuscript), or elaborate on this item by providing additional information not in the ms, or briefly explain why the item is not applicable/relevant for your study

"Objectives
This randomized controlled trial (RCT) investigates the acceptability of an ICBT-i treatment and assesses whether ICBT-i is more effective than internet-administrated applied relaxation (active control condition) in reducing insomnia symptoms (as measured by the Insomnia Severity Index) comorbid to chronic pain. In addition, this RCT investigates the effects of ICBT-i on sleep diary measures, pain intensity, anxiety, depression, pain-related disability, and perceived health. Negative effects were also monitored and reported. Our primary hypothesis is that, compared with an active control condition, ICBT-i would lead to greater reductions in insomnia symptoms at the end of treatment. We also hypothesized that ICBT-i would be superior to an active control condition in terms of improvement in symptom-related sleep diary measures, pain intensity, anxiety, depression, pain-related disability, and perceived health. In addition, the improvements gained from treatment are hypothesized to be maintained for 6 months after treatment."

METHODS

3a) Description of trial design (such as parallel, factorial) including allocation ratio

Does your paper address CONSORT subitem 3a? *

Copy and paste relevant sections from the manuscript (include quotes in quotation marks "like this" to indicate direct quotes from your manuscript), or elaborate on this item by providing additional information not in the ms, or briefly explain why the item is not applicable/relevant for your study

"Objectives
This randomized controlled trial (RCT) investigates the acceptability of an ICBT-i treatment and assesses whether ICBT-i is more effective than internet-administrated applied relaxation (active control condition) in reducing insomnia symptoms (as measured by the ISI) comorbid to chronic pain. In addition, this RCT investigates the effects of ICBT-i on sleep diary measures, pain intensity, anxiety, depression, pain-related disability, and perceived health"; "Our primary hypothesis is that compared with an active control condition, ICBT-i would lead to greater reductions in insomnia symptoms at the end of treatment. We also hypothesized that ICBT-i would be superior to an active control condition in terms of improvement in symptom-related sleep diary measures, pain intensity, anxiety, depression, pain-related disability, and perceived health. In addition, the improvements gained from treatment are hypothesized to be maintained for 6 months after treatment."

3b) Important changes to methods after trial commencement (such as eligibility criteria), with reasons

Does your paper address CONSORT subitem 3b? *

Copy and paste relevant sections from the manuscript (include quotes in quotation marks "like this" to indicate direct quotes from your manuscript), or elaborate on this item by providing additional information not in the ms, or briefly explain why the item is not applicable/relevant for your study

No major change was made, interventions were kept.

3b-i) Bug fixes, Downtimes, Content Changes

Bug fixes, Downtimes, Content Changes: ehealth systems are often dynamic systems. A description of changes to methods therefore also includes important changes made on the intervention or comparator during the trial (e.g., major bug fixes or changes in the functionality or content) (5-iii) and other "unexpected events" that may have influenced study design such as staff changes, system failures/downtimes, etc. [2].

	1	2	3	4	5	
subitem not at all important	(●)	○	○	○	○	essential

Rensa markering

Does your paper address subitem 3b-i?

Copy and paste relevant sections from the manuscript (include quotes in quotation marks "like this" to indicate direct quotes from your manuscript), or elaborate on this item by providing additional information not in the ms, or briefly explain why the item is not applicable/relevant for your study

Ditt svar

4a) Eligibility criteria for participants

Does your paper address CONSORT subitem 4a? *

Copy and paste relevant sections from the manuscript (include quotes in quotation marks "like this" to indicate direct quotes from your manuscript), or elaborate on this item by providing additional information not in the ms, or briefly explain why the item is not applicable/relevant for your study

"Participants with an ISI score of >14 (moderate to severe insomnia symptoms) and aged 18-65 years were asked to participate in the trial either by their physician or via postal invitation. Retrospective analyses were performed to identify former patients with an ISI score of >14 over the previous 2 years. If interested, participants registered on the website and completed another survey covering anxiety and depressive symptoms, demographic and physiological variables, current insomnia symptoms, pain characteristics, pain-related disability, and perceived health. Again, a cutoff score of >14 points on the ISI was used as an inclusion criterion. This measurement was considered as baseline. Participants were also asked to rate their average pain intensity during the previous week on a numeric rating scale as part of the baseline assessment. Finally, they were asked, "For how long have you had your pain problem?" The following predefined answers were provided: (1) less than 3 months, (2) 3 months to 1 year, (3) 1 to 3 years, (4) 3 to 5 years, or (5) >5 years. Participants who gave responses (2) to (5) were included in the study. When this strategy had been applied, the study sample consisted of 54 participants, and the decision was made to end recruitment and analyze that smaller sample.
When informed consent was provided, a staff member called the participant to set up a telephone interview within 5 days from registration on the website. The interview included the Mini International Neuropsychiatric Interview [32] (Swedish translation version 7.0.0) to cover psychiatric comorbidity and to identify bipolar disorder and psychoses, which were the absolute exclusion criteria. In addition, the telephone interview was used to evaluate whether the participant fulfilled the diagnostic criteria for insomnia disorder according to the Diagnostic and Statistical Manual of Mental Disorders, 5th Edition (Table S1 in Multimedia Appendix 2) [33] and to assess other inclusion and exclusion criteria. These criteria are detailed in Table S2 in Multimedia Appendix 2. All cases were evaluated by an inclusion and exclusion committee consisting of 2 experienced psychologists and 1 psychiatrist, when required."

4a-i) Computer / Internet literacy

Computer / Internet literacy is often an implicit “de facto” eligibility criterion - this should be explicitly clarified.

	1	2	3	4	5	
subitem not at all important	(selected)	○	○	○	○	essential

Rensa markering

Does your paper address subitem 4a-i?

Copy and paste relevant sections from the manuscript (include quotes in quotation marks "like this" to indicate direct quotes from your manuscript), or elaborate on this item by providing additional information not in the ms, or briefly explain why the item is not applicable/relevant for your study

Ditt svar

4a-ii) Open vs. closed, web-based vs. face-to-face assessments:

Open vs. closed, web-based vs. face-to-face assessments: Mention how participants were recruited (online vs. offline), e.g., from an open access website or from a clinic, and clarify if this was a purely web-based trial, or there were face-to-face components (as part of the intervention or for assessment), i.e., to what degree got the study team to know the participant. In online-only trials, clarify if participants were quasi-anonymous and whether having multiple identities was possible or whether technical or logistical measures (e.g., cookies, email confirmation, phone calls) were used to detect/prevent these.

	1	2	3	4	5	
subitem not at all important	○	○	◉	○	○	essential

Rensa markering

Does your paper address subitem 4a-ii? *

Copy and paste relevant sections from the manuscript (include quotes in quotation marks "like this" to indicate direct quotes from your manuscript), or elaborate on this item by providing additional information not in the ms, or briefly explain why the item is not applicable/relevant for your study

Ditt svar

4a-iii) Information giving during recruitment

Information given during recruitment. Specify how participants were briefed for recruitment and in the informed consent procedures (e.g., publish the informed consent documentation as appendix, see also item X26), as this information may have an effect on user self-selection, user expectation and may also bias results.

	1	2	3	4	5	
subitem not at all important	○	○	○	●	○	essential

Rensa markering

Does your paper address subitem 4a-iii?

Copy and paste relevant sections from the manuscript (include quotes in quotation marks "like this" to indicate direct quotes from your manuscript), or elaborate on this item by providing additional information not in the ms, or briefly explain why the item is not applicable/relevant for your study

"When informed consent was provided, a staff member called the participant to set up a telephone interview within 5 days from registration on the website"

4b) Settings and locations where the data were collected

Does your paper address CONSORT subitem 4b? *

Copy and paste relevant sections from the manuscript (include quotes in quotation marks "like this" to indicate direct quotes from your manuscript), or elaborate on this item by providing additional information not in the ms, or briefly explain why the item is not applicable/relevant for your study

"the Pain and Rehabilitation Centre, Linköping University Hospital, Sweden"

4b-i) Report if outcomes were (self-)assessed through online questionnaires

Clearly report if outcomes were (self-)assessed through online questionnaires (as common in web-based trials) or otherwise.

	1	2	3	4	5	
subitem not at all important	○	○	○	○	◉	essential

Rensa markering

Does your paper address subitem 4b-i? *

Copy and paste relevant sections from the manuscript (include quotes in quotation marks "like this" to indicate direct quotes from your manuscript), or elaborate on this item by providing additional information not in the ms, or briefly explain why the item is not applicable/relevant for your study

"Retrospective analyses were performed to identify former patients with an ISI score of >14 over the previous 2 years. If interested, participants registered on the website and completed another survey covering anxiety and depressive symptoms, demographic and physiological variables, current insomnia symptoms, pain characteristics, pain-related disability, and perceived health. Again, a cutoff score of >14 points on the ISI was used as an inclusion criterion"

4b-ii) Report how institutional affiliations are displayed

Report how institutional affiliations are displayed to potential participants [on ehealth media], as affiliations with prestigious hospitals or universities may affect volunteer rates, use, and reactions with regards to an intervention.(Not a required item – describe only if this may bias results)

	1	2	3	4	5	
subitem not at all important	◉	○	○	○	○	essential

Rensa markering

Does your paper address subitem 4b-ii?

Copy and paste relevant sections from the manuscript (include quotes in quotation marks "like this" to indicate direct quotes from your manuscript), or elaborate on this item by providing additional information not in the ms, or briefly explain why the item is not applicable/relevant for your study

Not stated in manuscript at all

5) The interventions for each group with sufficient details to allow replication, including how and when they were actually administered

5-i) Mention names, credential, affiliations of the developers, sponsors, and owners

Mention names, credential, affiliations of the developers, sponsors, and owners [6] (if authors/evaluators are owners or developer of the software, this needs to be declared in a "Conflict of interest" section or mentioned elsewhere in the manuscript).

	1	2	3	4	5	
subitem not at all important	○	○	●	○	○	essential

Rensa markering

Does your paper address subitem 5-i?

Copy and paste relevant sections from the manuscript (include quotes in quotation marks "like this" to indicate direct quotes from your manuscript), or elaborate on this item by providing additional information not in the ms, or briefly explain why the item is not applicable/relevant for your study

"The ICBT-i treatment conducted is a novel treatment developed by TW and PM and provided via the internet platform iterapi.se [34]. The treatment is based on the most well-established CBT principles for the treatment of insomnia (ie, sleep restriction and stimulus control) [35,36]."; "The internet-based control condition is a slightly modified version of the well-established applied relaxation techniques developed by Öst [38]. This method was chosen because it is a common treatment component that has a credible and applicable rationale for the treatment of both insomnia [39,40] and chronic pain [41,42]. Furthermore, applied relaxation is an active and rather time-consuming treatment that is supposed to control for time spent, measurement effects, and therapist support. The manual was adapted so that the length of treatment matched that of the experimental group (5 weeks)."

5-ii) Describe the history/development process

Describe the history/development process of the application and previous formative evaluations (e.g., focus groups, usability testing), as these will have an impact on adoption/use rates and help with interpreting results.

	1	2	3	4	5	
subitem not at all important	○	○	◉	○	○	essential

Rensa markering

Does your paper address subitem 5-ii?

Copy and paste relevant sections from the manuscript (include quotes in quotation marks "like this" to indicate direct quotes from your manuscript), or elaborate on this item by providing additional information not in the ms, or briefly explain why the item is not applicable/relevant for your study

"The internet-based control condition is a slightly modified
version of the well-established applied relaxation techniques
developed by Öst [38]. This method was chosen because it is a
common treatment component that has a credible and applicable
rationale for the treatment of both insomnia [39,40] and chronic
pain [41,42]. Furthermore, applied relaxation is an active and
rather time-consuming treatment that is supposed to control for
time spent, measurement effects, and therapist support. The
manual was adapted so that the length of treatment matched
that of the experimental group (5 weeks).", "The treatment is based on the most well-established CBT
principles for the treatment of insomnia (ie, sleep restriction
and stimulus control) [35,36]."

5-iii) Revisions and updating

Revisions and updating. Clearly mention the date and/or version number of the application/intervention (and comparator, if applicable) evaluated, or describe whether the intervention underwent major changes during the evaluation process, or whether the development and/or content was “frozen” during the trial. Describe dynamic components such as news feeds or changing content which may have an impact on the replicability of the intervention (for unexpected events see item 3b).

	1	2	3	4	5	
subitem not at all important	(•)	( )	( )	( )	( )	essential

Rensa markering

Does your paper address subitem 5-iii?

Copy and paste relevant sections from the manuscript (include quotes in quotation marks "like this" to indicate direct quotes from your manuscript), or elaborate on this item by providing additional information not in the ms, or briefly explain why the item is not applicable/relevant for your study

We did not change the treatment content during the trial

5-iv) Quality assurance methods

Provide information on quality assurance methods to ensure accuracy and quality of information provided [1], if applicable.

	1	2	3	4	5	
subitem not at all important	(●)	( )	( )	( )	( )	essential

Rensa markering

Does your paper address subitem 5-iv?

Copy and paste relevant sections from the manuscript (include quotes in quotation marks "like this" to indicate direct quotes from your manuscript), or elaborate on this item by providing additional information not in the ms, or briefly explain why the item is not applicable/relevant for your study

"The treatment is based on the most well-established CBT principles for the treatment of insomnia (i.e., sleep restriction and stimulus control)."

Further, theraspists were provided supervision to ensure treatment fidelity.

5-v) Ensure replicability by publishing the source code, and/or providing screenshots/screen-capture video, and/or providing flowcharts of the algorithms used

Ensure replicability by publishing the source code, and/or providing screenshots/screen-capture video, and/or providing flowcharts of the algorithms used. Replicability (i.e., other researchers should in principle be able to replicate the study) is a hallmark of scientific reporting.

	1	2	3	4	5	
subitem not at all important	(●)	○	○	○	○	essential

Rensa markering

Does your paper address subitem 5-v?

Copy and paste relevant sections from the manuscript (include quotes in quotation marks "like this" to indicate direct quotes from your manuscript), or elaborate on this item by providing additional information not in the ms, or briefly explain why the item is not applicable/relevant for your study

All treatment content is available (in swedish) from the authors on request.

5-vi) Digital preservation

Digital preservation: Provide the URL of the application, but as the intervention is likely to change or disappear over the course of the years; also make sure the intervention is archived (Internet Archive, webcitation.org, and/or publishing the source code or screenshots/videos alongside the article). As pages behind login screens cannot be archived, consider creating demo pages which are accessible without login.

	1	2	3	4	5	
subitem not at all important	(•)	( )	( )	( )	( )	essential

Rensa markering

Does your paper address subitem 5-vi?

Copy and paste relevant sections from the manuscript (include quotes in quotation marks "like this" to indicate direct quotes from your manuscript), or elaborate on this item by providing additional information not in the ms, or briefly explain why the item is not applicable/relevant for your study

"provided via the internet platform iterapi.se"

5-vii) Access

Access: Describe how participants accessed the application, in what setting/context, if they had to pay (or were paid) or not, whether they had to be a member of specific group. If known, describe how participants obtained “access to the platform and Internet” [1]. To ensure access for editors/reviewers/readers, consider to provide a “backdoor” login account or demo mode for reviewers/readers to explore the application (also important for archiving purposes, see vi).

	1	2	3	4	5	
subitem not at all important	○	◉	○	○	○	essential

Rensa markering

Does your paper address subitem 5-vii? *

Copy and paste relevant sections from the manuscript (include quotes in quotation marks "like this" to indicate direct quotes from your manuscript), or elaborate on this item by providing additional information not in the ms, or briefly explain why the item is not applicable/relevant for your study

They had to have chronic pain and issues with their sleep, and internet access which is obvious from the manuscript

5-viii) Mode of delivery, features/functionalities/components of the intervention and comparator, and the theoretical framework

Describe mode of delivery, features/functionalities/components of the intervention and comparator, and the theoretical framework [6] used to design them (instructional strategy [1], behaviour change techniques, persuasive features, etc., see e.g., [7, 8] for terminology). This includes an in-depth description of the content (including where it is coming from and who developed it) [1],” whether [and how] it is tailored to individual circumstances and allows users to track their progress and receive feedback” [6]. This also includes a description of communication delivery channels and – if computer-mediated communication is a component – whether communication was synchronous or asynchronous [6]. It also includes information on presentation strategies [1], including page design principles, average amount of text on pages, presence of hyperlinks to other resources, etc. [1].

	1	2	3	4	5	
subitem not at all important	○	○	○	◉	○	essential

Rensa markering

Does your paper address subitem 5-viii? *

Copy and paste relevant sections from the manuscript (include quotes in quotation marks "like this" to indicate direct quotes from your manuscript), or elaborate on this item by providing additional information not in the ms, or briefly explain why the item is not applicable/relevant for your study

"The rationale for the interventions and information about sleep were provided via SMS text messaging or PowerPoint (Microsoft Corp) presentations with speaker voice; participants were free to choose modality based on preference. Treatments were always initiated on Mondays, as the participants were gaining access to a new treatment module. They were instructed to hand in their home assignments the next Sunday to receive feedback or therapist support within 48 hours. Handing in-home assignments was mandatory to gain access to the next treatment module, and no treatment lasted longer than the agreed 5 weeks."; The ICBT-i treatment conducted is a novel treatment developed
by TW and PM and provided via the internet platform iterapi.se [34]. The treatment is based on the most well-established CBT principles for the treatment of insomnia (ie, sleep restriction and stimulus control) [35,36]. The first week focused on a short treatment rationale and registration of current sleep patterns (sleep diary). On the basis of the sleep diary data, an individually designed sleep prescription (bedtime) was calculated and applied during the second week. The prescription was equal to the calculated average total sleep time, but no shorter than 4 hours a night. The prescribed sleep time was maintained for the rest of the treatment unless the sleep efficiency exceeded 85% or sunk below 75%; then, the prescribed sleep time was adjusted to 15 to 30 minutes a week. High sleep efficiency resulted in more time in bed and low sleep efficiency resulted in less time in bed (although not <4 hours/night). The third module taught stimulus control, which is based on classical conditioning. Participants were told to use the bedroom only for sleep and sex. That is, activities such as watching television, reading, and social media consumption were not to be conducted in the bedroom. In addition, the participants were told to go to bed only when sleepy and to get out of bed and leave the bedroom

when unable to sleep. Similarly, the participants were told not to sleep in places other than the bedroom or outside the prescribed bedtimes. If stimulus control is implemented, it will restrict time in bed, similar to sleep restriction.

Week 4 was dedicated to daytime activity. Advice regarding activity balance was provided. This advice is based on the research conducted by Andrews et al [37], who found that patients with chronic pain who engage in irregular daytime activities experience poor sleep the same night. Daytime sleepiness can lead to inactivity; however, excessive activity can increase pain and hinder the ability to wind down before" "bedtime. The advice aims to facilitate a healthy level of daytime

activity to promote sleep and create a contrast between daytime activities and nighttime rest. The last week focused on maintaining behavior changes and preventing relapse. Generally, the aim is to keep the intervention as brief as possible while maintaining the main part of the treatment effect. Therefore, some usual CBT-i components were excluded, such as behavioral activation, scheduled worry time, and cognitive restructuring.

Applied Relaxation Control Condition

The internet-based control condition is a slightly modified version of the well-established applied relaxation techniques developed by Öst [38]. This method was chosen because it is a common treatment component that has a credible and applicable rationale for the treatment of both insomnia [39,40] and chronic pain [41,42]. Furthermore, applied relaxation is an active and rather time-consuming treatment that is supposed to control for time spent, measurement effects, and therapist support. The manual was adapted so that the length of treatment matched that of the experimental group (5 weeks). In addition to a short rationale and registration of current sleep patterns (sleep diary), the first week focused on progressive relaxation and diaphragmatic breathing. Participants were told to practice for 15 minutes twice a day and to keep a log for registration and evaluation. In the following week, time was shortened to 7 minutes twice a day. During the third week, participants were taught conditioned relaxation, and exercises were 2 to 3 minutes long. Differentiated relaxation aims only to activate the muscles required to perform a specific task (ie, other muscles can be

[illegible]
relaxed). This was the focus of the fourth week of the study. During the fifth week, participants were taught quick relaxation. As this technique can result in relaxation in just a few seconds, it can be applied several times throughout the day and at bedtime.
Therapist Support
Both treatment arms had therapist support every week of treatment. Support was provided via written messages in the treatment platform. Therapists (master's students in psychology or senior psychologists) provided problem solving and feedback on weekly tasks and ensured the correct implementation of treatment components. Because therapist support is one of the factors that contribute to treatment outcome [30], there was no restriction of therapist support, as it did not include components from the other treatment arm. The master's students were supervised by senior psychologists trained in CBT (TW and PM). In a few cases, telephone calls were used to solve technical problems or to reach participants who did not respond to written messages. The same therapists provided both treatments, and the participants were distributed because of the randomization. Because the treatment content was standardized in both treatment arms, no measures of therapist fidelity were collected"

5-ix) Describe use parameters

Describe use parameters (e.g., intended "doses" and optimal timing for use). Clarify what instructions or recommendations were given to the user, e.g., regarding timing, frequency, heaviness of use, if any, or was the intervention used ad libitum.

	1	2	3	4	5	
subitem not at all important	○	○	○	●	○	essential

Rensa markering

Does your paper address subitem 5-ix?

Copy and paste relevant sections from the manuscript (include quotes in quotation marks "like this" to indicate direct quotes from your manuscript), or elaborate on this item by providing additional information not in the ms, or briefly explain why the item is not applicable/relevant for your study

"Both treatment arms had therapist support every week of treatment. Support was provided via written messages in the treatment platform. Therapists (master's students in psychology or senior psychologists) provided problem solving and feedback on weekly tasks and ensured the correct implementation of treatment components. Because therapist support is one of the factors that contribute to treatment outcome [30], there was no restriction of therapist support, as it did not include components from the other treatment arm. The master's students were supervised by senior psychologists trained in CBT (TW and PM). In a few cases, telephone calls were used to solve technical problems or to reach participants who did not respond to written messages"

5-x) Clarify the level of human involvement

Clarify the level of human involvement (care providers or health professionals, also technical assistance) in the e-intervention or as co-intervention (detail number and expertise of professionals involved, if any, as well as "type of assistance offered, the timing and frequency of the support, how it is initiated, and the medium by which the assistance is delivered". It may be necessary to distinguish between the level of human involvement required for the trial, and the level of human involvement required for a routine application outside of a RCT setting (discuss under item 21 – generalizability).

	1	2	3	4	5	
subitem not at all important	○	○	○	●	○	essential

Rensa markering

Does your paper address subitem 5-x?

Copy and paste relevant sections from the manuscript (include quotes in quotation marks "like this" to indicate direct quotes from your manuscript), or elaborate on this item by providing additional information not in the ms, or briefly explain why the item is not applicable/relevant for your study

"Both treatment arms had therapist support every week of treatment. Support was provided via written messages in the treatment platform."; In a few cases, telephone calls were used to solve technical problems or to reach participants who did not respond to written messages."; "When informed consent was provided, a staff member called the participant to set up a telephone interview within 5 days from registration on the website"; "Participants with an ISI score of >14 (moderate to severe insomnia symptoms) and aged 18-65 years were asked to participate in the trial either by their physician or via postal invitation."

5-xi) Report any prompts/reminders used

Report any prompts/reminders used: Clarify if there were prompts (letters, emails, phone calls, SMS) to use the application, what triggered them, frequency etc. It may be necessary to distinguish between the level of prompts/reminders required for the trial, and the level of prompts/reminders for a routine application outside of a RCT setting (discuss under item 21 – generalizability).

	1	2	3	4	5	
subitem not at all important	○	○	○	○	○	essential

Does your paper address subitem 5-xi? *

Copy and paste relevant sections from the manuscript (include quotes in quotation marks "like this" to indicate direct quotes from your manuscript), or elaborate on this item by providing additional information not in the ms, or briefly explain why the item is not applicable/relevant for your study

E-mail was used to remind subjects to complete treatment modules if necessary and telephone support was only used to solve technical issues.

5-xii) Describe any co-interventions (incl. training/support)

Describe any co-interventions (incl. training/support): Clearly state any interventions that are provided in addition to the targeted eHealth intervention, as ehealth intervention may not be designed as stand-alone intervention. This includes training sessions and support [1]. It may be necessary to distinguish between the level of training required for the trial, and the level of training for a routine application outside of a RCT setting (discuss under item 21 – generalizability.

	1	2	3	4	5	
subitem not at all important	◉	○	○	○	○	essential

Rensa markering

Does your paper address subitem 5-xii? *

Copy and paste relevant sections from the manuscript (include quotes in quotation marks "like this" to indicate direct quotes from your manuscript), or elaborate on this item by providing additional information not in the ms, or briefly explain why the item is not applicable/relevant for your study

There were no other targets of the intervention per se other than sleep, and change in secondary measures only envisioned as a consequence of that.

6a) Completely defined pre-specified primary and secondary outcome measures, including how and when they were assessed

Does your paper address CONSORT subitem 6a? *

Copy and paste relevant sections from the manuscript (include quotes in quotation marks "like this" to indicate direct quotes from your manuscript), or elaborate on this item by providing additional information not in the ms, or briefly explain why the item is not applicable/relevant for your study

"Primary Outcome: Insomnia Severity
The Swedish version of the ISI was used to quantify perceived insomnia severity. The ISI, a valid instrument with excellent internal consistency, captured the severity and impact of insomnia symptoms [5,43"; "Secondary Outcomes
Sleep Parameters Assessed by Responsive Sleep Diary
All participants were asked to complete a sleep diary throughout the treatment and for 1 week at the 6-month follow-up. These data were used to calculate the sleep-specific outcomes, that is, sleep onset latency (SOL), wake time after sleep onset (WASO), time in bed, sleep efficiency, and early morning awakenings (EMA), according to Buysse et al [45]. Every measure is based on the mean value form all registered nights that week. The patient interface presents the time in bed, total sleep time, and sleep efficiency for every night and the mean values for every week.
Sleepiness
Sleepiness was measured using the Karolinska Sleepiness Scale (KSS), which is a 9-graded scale that measures the level of sleepiness at a particular time during the day [46]. Every other step is labeled as follows: 1=very alert, 3=alert, 5=neither alert nor sleepy, 7=sleepy (but not fighting sleep), and 9=very sleepy (fighting sleep). The KSS is correlated with several electroencephalogram measures (eg, α-activity, r=0.40) and behavior variables (eg, mean reaction time on the psychomotor vigilance task, r=0.57) related to sleepiness. As test-retest reliability depends on the time of day, the KSS was sent to participants via SMS text messaging at 11 AM every day during treatment. If the participants did not answer the question within 60 minutes of receiving the SMS text message, the value was reported as missing. The KSS was added to the sleep diary to capture nonrestorative sleep and daytime symptoms.
Anxiety

The Generalized Anxiety Disorder–7-item (GAD-7) scale was used to measure anxiety symptoms [47]. The GAD-7 measures symptoms of generalized anxiety disorder but is also highly correlated with more general anxiety measures [47]. Each itemhas an answer response ranging from 0 to 3 and is scored with
respect to the frequency of the symptom during the previous 2 weeks: not at all, several days, more than half the days, and nearly every day. This gives a total score of 0 to 21. Optimal sensitivity/specificity ratio for detecting generalized anxiety disorder is obtained with a cutoff score of ≥10. The GAD-7 has shown excellent internal consistency (Cronbach α=.92) [47]. The Cronbach α for the GAD-7 in our sample was .88.

Depression

The Patient Health Questionnaire–9 items (PHQ-9) was used to measure depression [48].
The PHQ-9 is a 9-item self-rating scale reflecting the Diagnostic and Statistical Manual of Mental Disorders, 4th Edition, criteria for major depressive disorder [48]. Each item ranges from 0 to 3 points and is scored with respect to the frequency of the symptom during the last 2 weeks: not at all, several days, more than half the days, and nearly every day. This gives a total score of 0 to 27. The PHQ-9 has shown excellent internal consistency (Cronbach α=.86-.89) [48] and strong correlations with other well-established depression scales such as the Beck Depression Inventory II (r=0.84) and the Montgomery-Åsberg Depression Rating Scale (r=0.79) [49]. The Cronbach α for the PHQ-9 in our sample was .82. Optimal sensitivity/specificity ratio for detecting depression is obtained with a cutoff score of ≥10.

Pain Intensity

The participants were asked to rate their average pain intensity during the previous week on a numeric rating scale ranging from 0 (no pain) to 10 (worst imaginable pain). This scale has provided good validity in experimental conditions [50].

Number of Anatomical Pain Regions

The participants were presented with a list of 36 anatomical regions covering the entire body and were asked to mark all regions where they experienced pain. A total score index was calculated (total score=0-36) [51].

Pain Disability

The Pain Disability Index (PDI) was used to measure the

The Pain Disability Index (PDI) was used to measure the self-reported pain-related disability [52]. The PDI has high internal consistency (Cronbach α=.85) in patients seeking specialized pain care [53]. Minimal important change depends on baseline scores [54] (eg, baseline values 28-42 require a decrease of at least 15 points). The Cronbach α for the PDI in our sample was .81.

Health-Related Quality of Life

The Health Visual Analog Scale from the European Quality of Life instrument was used to measure the current state of self-estimated health [55]. The item consists of a 100-point thermometer-like vertical scale with defined end points (worst imaginable health condition and best imaginable health condition). Higher values indicate better health, whereas lower values indicate worse health [55,56].

Potentially Adverse and Unwanted Events

The Negative Effects Questionnaire (NEQ), used to assess the side effects of psychological treatment, consists of 32 items with three parts [57]: an initial yes or no question regarding the occurrence of the negative effect in question and a rating of the impact of the negative effect using a 5-point scale (not at all to extremely). The patient also judges whether the negative effect was caused by the treatment. In this RCT, the NEQ was used to screen for and quantify the most common negative effects attributed to the treatment. There is no consensus in the scientific community on how to present results for negative effects, as definitions and measures vary. Generally, it is recommended to provide the frequencies of the negative effects that have occurred the most. The NEQ has good internal consistency (α=.95) and six factors: symptoms, quality, dependency, stigma, hopelessness, and failure [58].

The Cronbach α for the NEQ in our sample was .80."

6a-i) Online questionnaires: describe if they were validated for online use and apply CHERRIES items to describe how the questionnaires were designed/deployed

If outcomes were obtained through online questionnaires, describe if they were validated for online use and apply CHERRIES items to describe how the questionnaires were designed/deployed [9].

	1	2	3	4	5	
subitem not at all important	○	○	○	○	○	essential

Does your paper address subitem 6a-i?

Copy and paste relevant sections from manuscript text

Ditt svar

6a-ii) Describe whether and how “use” (including intensity of use/dosage) was defined/measured/monitored

Describe whether and how “use” (including intensity of use/dosage) was defined/measured/monitored (logins, logfile analysis, etc.). Use/adoption metrics are important process outcomes that should be reported in any ehealth trial.

	1	2	3	4	5	
subitem not at all important	○	○	◉	○	○	essential

Rensa markering

Does your paper address subitem 6a-ii?

Copy and paste relevant sections from manuscript text

"The number of treatment modules with submitted tasks or worksheets was reported as median values and their IQR. Modules with submitted tasks or worksheets were used as proxies for treatment compliance. This definition did not require full completion of all tasks or worksheets of a module, and any answer (in addition to a sleep diary) was interpreted as a result of working with the treatment content from that module"; "Both treatment arms were provided with weekly home assignments"

6a-iii) Describe whether, how, and when qualitative feedback from participants was obtained

Describe whether, how, and when qualitative feedback from participants was obtained (e.g., through emails, feedback forms, interviews, focus groups).

	1	2	3	4	5	
subitem not at all important	○	●	○	○	○	essential

Rensa markering

Does your paper address subitem 6a-iii?

Copy and paste relevant sections from manuscript text

Qualitative feedback was obtained at the end of the last module in order to improve future studies.

6b) Any changes to trial outcomes after the trial commenced, with reasons

Does your paper address CONSORT subitem 6b? *

Copy and paste relevant sections from the manuscript (include quotes in quotation marks "like this" to indicate direct quotes from your manuscript), or elaborate on this item by providing additional information not in the ms, or briefly explain why the item is not applicable/relevant for your study

No, it did not change in regards to outcomes

7a) How sample size was determined

NPT: When applicable, details of whether and how the clustering by care provides or centers was addressed

7a-i) Describe whether and how expected attrition was taken into account when calculating the sample size

Describe whether and how expected attrition was taken into account when calculating the sample size.

	1	2	3	4	5	
subitem not at all important	○	○	○	◉	○	essential

Rensa markering

Does your paper address subitem 7a-i?

Copy and paste relevant sections from manuscript title (include quotes in quotation marks "like this" to indicate direct quotes from your manuscript), or elaborate on this item by providing additional information not in the ms, or briefly explain why the item is not applicable/relevant for your study

"An estimated effect
size of 0.6 and an α of .05, gave a sample of at least 40
participants per study condition [27,31]. An anticipated dropout
of one-third resulted in the expected need for a total sample of
120 participants."

7b) When applicable, explanation of any interim analyses and stopping guidelines

Does your paper address CONSORT subitem 7b? *

Copy and paste relevant sections from the manuscript (include quotes in quotation marks "like this" to indicate direct quotes from your manuscript), or elaborate on this item by providing additional information not in the ms, or briefly explain why the item is not applicable/relevant for your study

No such analysis or stopping guide lines were used.

8a) Method used to generate the random allocation sequence

NPT: When applicable, how care providers were allocated to each trial group

Does your paper address CONSORT subitem 8a? *

Copy and paste relevant sections from the manuscript (include quotes in quotation marks "like this" to indicate direct quotes from your manuscript), or elaborate on this item by providing additional information not in the ms, or briefly explain why the item is not applicable/relevant for your study

"Included participants were randomized via random.org in blocks of 34 participants to either the ICBT-i group or the applied relaxation group."

8b) Type of randomisation; details of any restriction (such as blocking and block size)

Does your paper address CONSORT subitem 8b? *

Copy and paste relevant sections from the manuscript (include quotes in quotation marks "like this" to indicate direct quotes from your manuscript), or elaborate on this item by providing additional information not in the ms, or briefly explain why the item is not applicable/relevant for your study

"Included participants were randomized via random.org in blocks of 34 participants to either the ICBT-i group or the applied relaxation group. This procedure was performed to ensure equal numbers of participants in each arm after the first postal survey. Thereafter, participants were added to a randomized list consecutively (ensuring an equal sample size after 34 additional participants)."

9) Mechanism used to implement the random allocation sequence (such as sequentially numbered containers), describing any steps taken to conceal the sequence until interventions were assigned

Does your paper address CONSORT subitem 9? *

Copy and paste relevant sections from the manuscript (include quotes in quotation marks "like this" to indicate direct quotes from your manuscript), or elaborate on this item by providing additional information not in the ms, or briefly explain why the item is not applicable/relevant for your study

"Participants were blinded to the experiment or control and were told that they were assigned to 1 of the 2 treatments. Allocation was conducted after the inclusion and exclusion committee met, and participants were notified the week before treatment started. The therapists were not blinded."

10) Who generated the random allocation sequence, who enrolled participants, and who assigned participants to interventions

Does your paper address CONSORT subitem 10? *

Copy and paste relevant sections from the manuscript (include quotes in quotation marks "like this" to indicate direct quotes from your manuscript), or elaborate on this item by providing additional information not in the ms, or briefly explain why the item is not applicable/relevant for your study

A fellow researcher, not otherwise involved in this study, generated the random allocation sequence in blocks of 34 subjects. Included subjects were then added to this list (by TW) in the order they appliedfor the study. Enrollment was cunducted by master students in psychology or licenced psychologists.

11a) If done, who was blinded after assignment to interventions (for example, participants, care providers, those assessing outcomes) and how

NPT: Whether or not administering co-interventions were blinded to group assignment

11a-i) Specify who was blinded, and who wasn't

Specify who was blinded, and who wasn't. Usually, in web-based trials it is not possible to blind the participants [1, 3] (this should be clearly acknowledged), but it may be possible to blind outcome assessors, those doing data analysis or those administering co-interventions (if any).

	1	2	3	4	5	
subitem not at all important	○	○	○	◉	○	essential

Rensa markering

Does your paper address subitem 11a-i? *

Copy and paste relevant sections from the manuscript (include quotes in quotation marks "like this" to indicate direct quotes from your manuscript), or elaborate on this item by providing additional information not in the ms, or briefly explain why the item is not applicable/relevant for your study

"Participants were blinded to the experiment or control... "; "The therapists were not blinded."

11a-ii) Discuss e.g., whether participants knew which intervention was the “intervention of interest” and which one was the “comparator”

Informed consent procedures (4a-ii) can create biases and certain expectations - discuss e.g., whether participants knew which intervention was the “intervention of interest” and which one was the “comparator”.

	1	2	3	4	5	
subitem not at all important	○	○	○	●	○	essential

Rensa markering

Does your paper address subitem 11a-ii?

Copy and paste relevant sections from the manuscript (include quotes in quotation marks "like this" to indicate direct quotes from your manuscript), or elaborate on this item by providing additional information not in the ms, or briefly explain why the item is not applicable/relevant for your study

"were told that they were assigned to 1 of the 2 treatments."

11b) If relevant, description of the similarity of interventions

(this item is usually not relevant for ehealth trials as it refers to similarity of a placebo or sham intervention to a active medication/intervention)

Does your paper address CONSORT subitem 11b? *

Copy and paste relevant sections from the manuscript (include quotes in quotation marks "like this" to indicate direct quotes from your manuscript), or elaborate on this item by providing additional information not in the ms, or briefly explain why the item is not applicable/relevant for your study

As described in more detail previously, both are based on established psycholigcal treatments but can not be seen as variations on the same theme as their mechanisms are from two different theories.

12a) Statistical methods used to compare groups for primary and secondary outcomes

NPT: When applicable, details of whether and how the clustering by care providers or centers was addressed

Does your paper address CONSORT subitem 12a? *

Copy and paste relevant sections from the manuscript (include quotes in quotation marks "like this" to indicate direct quotes from your manuscript), or elaborate on this item by providing additional information not in the ms, or briefly explain why the item is not applicable/relevant for your study

"To examine the immediate (week 5) and long-term (6-month follow-up) treatment effects on the ISI and secondary outcomes, separate mixed models for repeated measures with treatment, time, and the interaction of treatment by time were performed with random intercept and random slope (whenever appropriate) using an unstructured covariance matrix. The examination of the variance of the slope for time was significant; therefore, a model with random slopes for time with multiple time points fitted the data, and a model with fixed slopes was more appropriate for time with 2 time points. Time was modeled as a numeric variable (0-4 and 0-1), and both linear and quadratic trajectories were considered; the latter was chosen when modeling revealed a significant trajectory"

12a-i) Imputation techniques to deal with attrition / missing values

Imputation techniques to deal with attrition / missing values: Not all participants will use the intervention/comparator as intended and attrition is typically high in ehealth trials. Specify how participants who did not use the application or dropped out from the trial were treated in the statistical analysis (a complete case analysis is strongly discouraged, and simple imputation techniques such as LOCF may also be problematic [4]).

	1	2	3	4	5	
subitem not at all important	○	○	○	○	◉	essential

Rensa markering

Does your paper address subitem 12a-i? *

Copy and paste relevant sections from the manuscript (include quotes in quotation marks "like this" to indicate direct quotes from your manuscript), or elaborate on this item by providing additional information not in the ms, or briefly explain why the item is not applicable/relevant for your study

"Minor
corrections were applied to the sleep diary data to account for
missing data and input errors: missing durations (but not
timestamps) for otherwise filled entries were replaced with
zeros; bed and sleep time were ordered so that the former always
came before the latter; k=9 entries with negative sleep time (primarily due to mathematically impossible wake durations)
were omitted; and k=8 entries obviously incorrectly inputted as
timestamps were manually recoded as durations."

"All available time points were included in the models. On the basis of the principle that data were missing at random, all linear mixed models were run with restricted maximum likelihood estimation, which can produce unbiased estimators."

12b) Methods for additional analyses, such as subgroup analyses and adjusted analyses

Does your paper address CONSORT subitem 12b? *

Copy and paste relevant sections from the manuscript (include quotes in quotation marks "like this" to indicate direct quotes from your manuscript), or elaborate on this item by providing additional information not in the ms, or briefly explain why the item is not applicable/relevant for your study

No subgroup-, or analysis adjusting for other variables, were conducted.

X26) REB/IRB Approval and Ethical Considerations [recommended as subheading under "Methods"] (not a CONSORT item)

X26-i) Comment on ethics committee approval

	1	2	3	4	5	
subitem not at all important	○	○	○	●	○	essential

Rensa markering

Does your paper address subitem X26-i?

Copy and paste relevant sections from the manuscript (include quotes in quotation marks "like this" to indicate direct quotes from your manuscript), or elaborate on this item by providing additional information not in the ms, or briefly explain why the item is not applicable/relevant for your study

"The study was approved by the ethical review board in Linköping (dnr. 2014/191-31). An additional application to the ethical committee was submitted (dnr. 2017/511-32) to enable invitation of patients further back in time. The participants also provided informed consent as directed by the World Medical Association Declaration of Helsinki."

x26-ii) Outline informed consent procedures

Outline informed consent procedures e.g., if consent was obtained offline or online (how? Checkbox, etc.?), and what information was provided (see 4a-ii). See [6] for some items to be included in informed consent documents.

	1	2	3	4	5	
subitem not at all important	○	○	○	◉	○	essential

Rensa markering

Does your paper address subitem X26-ii?

Copy and paste relevant sections from the manuscript (include quotes in quotation marks "like this" to indicate direct quotes from your manuscript), or elaborate on this item by providing additional information not in the ms, or briefly explain why the item is not applicable/relevant for your study

"They also provided informed consent as directed by the World Medical Association Declaration of Helsinki."

This was done online by writing their full names in a free text box.

X26-iii) Safety and security procedures

Safety and security procedures, incl. privacy considerations, and any steps taken to reduce the likelihood or detection of harm (e.g., education and training, availability of a hotline)

	1	2	3	4	5	
subitem not at all important	○	○	○	◉	○	essential

Rensa markering

Does your paper address subitem X26-iii?

Copy and paste relevant sections from the manuscript (include quotes in quotation marks "like this" to indicate direct quotes from your manuscript), or elaborate on this item by providing additional information not in the ms, or briefly explain why the item is not applicable/relevant for your study

Subjects needed a SMS-code to logg in on the treatment platform.

RESULTS

13a) For each group, the numbers of participants who were randomly assigned, received intended treatment, and were analysed for the primary outcome

NPT: The number of care providers or centers performing the intervention in each group and the number of patients treated by each care provider in each center

Does your paper address CONSORT subitem 13a? *

Copy and paste relevant sections from the manuscript (include quotes in quotation marks "like this" to indicate direct quotes from your manuscript), or elaborate on this item by providing additional information not in the ms, or briefly explain why the item is not applicable/relevant for your study

"At the
6-month follow-up, 77% (23/30) of the participants in the
ICBT-i group and 63% (15/24) of the participants in applied
relaxation group (ie, the control group) responded." All included participants were used in the mixed models analysis.

13b) For each group, losses and exclusions after randomisation, together with reasons

Does your paper address CONSORT subitem 13b? (NOTE: Preferably, this is shown in a CONSORT flow diagram) *

Copy and paste relevant sections from the manuscript (include quotes in quotation marks "like this" to indicate direct quotes from your manuscript), or elaborate on this item by providing additional information not in the ms, or briefly explain why the item is not applicable/relevant for your study

Yes, see the flow diagram (Figure 1)

13b-i) Attrition diagram

Strongly recommended: An attrition diagram (e.g., proportion of participants still logging in or using the intervention/comparator in each group plotted over time, similar to a survival curve) or other figures or tables demonstrating usage/dose/engagement.

	1	2	3	4	5	
subitem not at all important	○	○	○	○	○	essential

Does your paper address subitem 13b-i?

Copy and paste relevant sections from the manuscript or cite the figure number if applicable (include quotes in quotation marks "like this" to indicate direct quotes from your manuscript), or elaborate on this item by providing additional information not in the ms, or briefly explain why the item is not applicable/relevant for your study

Ditt svar

14a) Dates defining the periods of recruitment and follow-up

Does your paper address CONSORT subitem 14a? *

Copy and paste relevant sections from the manuscript (include quotes in quotation marks "like this" to indicate direct quotes from your manuscript), or elaborate on this item by providing additional information not in the ms, or briefly explain why the item is not applicable/relevant for your study

The treatments were five weeks long, and the study was concluded when 6 months had passed and the follow-up data was collected.

14a-i) Indicate if critical “secular events” fell into the study period

Indicate if critical “secular events” fell into the study period, e.g., significant changes in Internet resources available or “changes in computer hardware or Internet delivery resources”

	1	2	3	4	5	
subitem not at all important	○	○	○	○	○	essential

Does your paper address subitem 14a-i?

Copy and paste relevant sections from the manuscript (include quotes in quotation marks "like this" to indicate direct quotes from your manuscript), or elaborate on this item by providing additional information not in the ms, or briefly explain why the item is not applicable/relevant for your study

Ditt svar

14b) Why the trial ended or was stopped (early)

Does your paper address CONSORT subitem 14b? *

Copy and paste relevant sections from the manuscript (include quotes in quotation marks "like this" to indicate direct quotes from your manuscript), or elaborate on this item by providing additional information not in the ms, or briefly explain why the item is not applicable/relevant for your study

It ended when all participants who had not dropped out had be given a chance to answer the 6-month follow up

15) A table showing baseline demographic and clinical characteristics for each group

NPT: When applicable, a description of care providers (case volume, qualification, expertise, etc.) and centers (volume) in each group

Does your paper address CONSORT subitem 15? *

Copy and paste relevant sections from the manuscript (include quotes in quotation marks "like this" to indicate direct quotes from your manuscript), or elaborate on this item by providing additional information not in the ms, or briefly explain why the item is not applicable/relevant for your study

Yes, in table 1.

15-i) Report demographics associated with digital divide issues

In ehealth trials it is particularly important to report demographics associated with digital divide issues, such as age, education, gender, social-economic status, computer/Internet/ehealth literacy of the participants, if known.

	1	2	3	4	5	
subitem not at all important	○	○	◉	○	○	essential

Rensa markering

Does your paper address subitem 15-i? *

Copy and paste relevant sections from the manuscript (include quotes in quotation marks "like this" to indicate direct quotes from your manuscript), or elaborate on this item by providing additional information not in the ms, or briefly explain why the item is not applicable/relevant for your study

Age, education, gender are visible in table 1.

16) For each group, number of participants (denominator) included in each analysis and whether the analysis was by original assigned groups

16-i) Report multiple “denominators” and provide definitions

Report multiple “denominators” and provide definitions: Report N’s (and effect sizes) “across a range of study participation [and use] thresholds” [1], e.g., N exposed, N consented, N used more than x times, N used more than y weeks, N participants “used” the intervention/comparator at specific pre-defined time points of interest (in absolute and relative numbers per group). Always clearly define “use” of the intervention.

	1	2	3	4	5	
subitem not at all important	○	○	◉	○	○	essential

Rensa markering

Does your paper address subitem 16-i? *

Copy and paste relevant sections from the manuscript (include quotes in quotation marks "like this" to indicate direct quotes from your manuscript), or elaborate on this item by providing additional information not in the ms, or briefly explain why the item is not applicable/relevant for your study

Yes, stated with corresponding analysis.

16-ii) Primary analysis should be intent-to-treat

Primary analysis should be intent-to-treat, secondary analyses could include comparing only "users", with the appropriate caveats that this is no longer a randomized sample (see 18-i).

	1	2	3	4	5	
subitem not at all important	○	○	○	●	○	essential

Rensa markering

Does your paper address subitem 16-ii?

Copy and paste relevant sections from the manuscript (include quotes in quotation marks "like this" to indicate direct quotes from your manuscript), or elaborate on this item by providing additional information not in the ms, or briefly explain why the item is not applicable/relevant for your study

Yes, "The analysis was based on the intention-to-treat principles and the assumption that data were missing at random."

17a) For each primary and secondary outcome, results for each group, and the estimated effect size and its precision (such as 95% confidence interval)

Does your paper address CONSORT subitem 17a? *

Copy and paste relevant sections from the manuscript (include quotes in quotation marks "like this" to indicate direct quotes from your manuscript), or elaborate on this item by providing additional information not in the ms, or briefly explain why the item is not applicable/relevant for your study

Ditt svar

17a-i) Presentation of process outcomes such as metrics of use and intensity of use

In addition to primary/secondary (clinical) outcomes, the presentation of process outcomes such as metrics of use and intensity of use (dose, exposure) and their operational definitions is critical. This does not only refer to metrics of attrition (13-b) (often a binary variable), but also to more continuous exposure metrics such as “average session length”. These must be accompanied by a technical description how a metric like a “session” is defined (e.g., timeout after idle time) [1] (report under item 6a).

	1	2	3	4	5	
subitem not at all important	○	○	○	◉	○	essential

Rensa markering

Does your paper address subitem 17a-i?

Copy and paste relevant sections from the manuscript (include quotes in quotation marks "like this" to indicate direct quotes from your manuscript), or elaborate on this item by providing additional information not in the ms, or briefly explain why the item is not applicable/relevant for your study

Like this from Figure 3 "-0.48 [-1.07, 0.10]

17b) For binary outcomes, presentation of both absolute and relative effect sizes is recommended

Does your paper address CONSORT subitem 17b? *

Copy and paste relevant sections from the manuscript (include quotes in quotation marks "like this" to indicate direct quotes from your manuscript), or elaborate on this item by providing additional information not in the ms, or briefly explain why the item is not applicable/relevant for your study

No binary outcomes was used.

18) Results of any other analyses performed, including subgroup analyses and adjusted analyses, distinguishing pre-specified from exploratory

Does your paper address CONSORT subitem 18? *

Copy and paste relevant sections from the manuscript (include quotes in quotation marks "like this" to indicate direct quotes from your manuscript), or elaborate on this item by providing additional information not in the ms, or briefly explain why the item is not applicable/relevant for your study

No subgroup or adjusted analyses were performed

18-i) Subgroup analysis of comparing only users

A subgroup analysis of comparing only users is not uncommon in ehealth trials, but if done, it must be stressed that this is a self-selected sample and no longer an unbiased sample from a randomized trial (see 16-iii).

	1	2	3	4	5	
subitem not at all important	◉	○	○	○	○	essential

Rensa markering

Does your paper address subitem 18-i?

Copy and paste relevant sections from the manuscript (include quotes in quotation marks "like this" to indicate direct quotes from your manuscript), or elaborate on this item by providing additional information not in the ms, or briefly explain why the item is not applicable/relevant for your study

This sort of analysis is not part of the manuscript

19) All important harms or unintended effects in each group

(for specific guidance see CONSORT for harms)

Does your paper address CONSORT subitem 19? *

Copy and paste relevant sections from the manuscript (include quotes in quotation marks "like this" to indicate direct quotes from your manuscript), or elaborate on this item by providing additional information not in the ms, or briefly explain why the item is not applicable/relevant for your study

Yes, "The most frequently reported negative effect in both the treatment arms was increased stress. In the ICBT-i group, the second most reported negative effect was the experience that the treatment did not suit the patient. In contrast, the second most reported negative effect in the applied relaxation control condition was that participants stopped believing that there was available help. Table S9 in Multimedia Appendix 2 provides a comprehensive description of the negative effects."

19-i) Include privacy breaches, technical problems

Include privacy breaches, technical problems. This does not only include physical “harm” to participants, but also incidents such as perceived or real privacy breaches [1], technical problems, and other unexpected/unintended incidents. “Unintended effects” also includes unintended positive effects [2].

	1	2	3	4	5	
subitem not at all important	(●)	○	○	○	○	essential

Rensa markering

Does your paper address subitem 19-i?

Copy and paste relevant sections from the manuscript (include quotes in quotation marks "like this" to indicate direct quotes from your manuscript), or elaborate on this item by providing additional information not in the ms, or briefly explain why the item is not applicable/relevant for your study

No such major events were recorded. Only that some participants needed extra telephone guidance to get started.

19-ii) Include qualitative feedback from participants or observations from staff/researchers

Include qualitative feedback from participants or observations from staff/researchers, if available, on strengths and shortcomings of the application, especially if they point to unintended/unexpected effects or uses. This includes (if available) reasons for why people did or did not use the application as intended by the developers.

	1	2	3	4	5	
subitem not at all important	○	◉	○	○	○	essential

Rensa markering

Does your paper address subitem 19-ii?

Copy and paste relevant sections from the manuscript (include quotes in quotation marks "like this" to indicate direct quotes from your manuscript), or elaborate on this item by providing additional information not in the ms, or briefly explain why the item is not applicable/relevant for your study

Qualitative feedback was obtained at the end of the last module in order to improve future studies but this information was not part of this ms.

DISCUSSION

22) Interpretation consistent with results, balancing benefits and harms, and considering other relevant evidence

NPT: In addition, take into account the choice of the comparator, lack of or partial blinding, and unequal expertise of care providers or centers in each group

22-i) Restate study questions and summarize the answers suggested by the data, starting with primary outcomes and process outcomes (use)

Restate study questions and summarize the answers suggested by the data, starting with primary outcomes and process outcomes (use).

	1	2	3	4	5	
subitem not at all important	○	○	○	◉	○	essential

Rensa markering

Does your paper address subitem 22-i? *

Copy and paste relevant sections from the manuscript (include quotes in quotation marks "like this" to indicate direct quotes from your manuscript), or elaborate on this item by providing additional information not in the ms, or briefly explain why the item is not applicable/relevant for your study

"Our results suggest that ICBT-i decreases insomnia severity and related nighttime symptoms. In particular, we found that the ICBT-i group, compared with the applied relaxation group, had greater immediate improvements in insomnia symptoms as measured by ISI, WASO, sleep efficiency, and EMA. The immediate effect (within-subject) of ICBT-i on ISI was clinically significant according to definition by Morin et al [5]. After 6 months, the group means approached each other, and no differences were confirmed on ISI (or other sleep parameters)."

22-ii) Highlight unanswered new questions, suggest future research

Highlight unanswered new questions, suggest future research.

	1	2	3	4	5	
subitem not at all important	○	○	○	◉	○	essential

Rensa markering

Does your paper address subitem 22-ii?

Copy and paste relevant sections from the manuscript (include quotes in quotation marks "like this" to indicate direct quotes from your manuscript), or elaborate on this item by providing additional information not in the ms, or briefly explain why the item is not applicable/relevant for your study

"However, further research is needed to
investigate the effect of ICBT-i on a larger sample size of people
with chronic pain."

20) Trial limitations, addressing sources of potential bias, imprecision, and, if relevant, multiplicity of analyses

20-i) Typical limitations in ehealth trials

Typical limitations in ehealth trials: Participants in ehealth trials are rarely blinded. Ehealth trials often look at a multiplicity of outcomes, increasing risk for a Type I error. Discuss biases due to non-use of the intervention/usability issues, biases through informed consent procedures, unexpected events.

	1	2	3	4	5	
subitem not at all important	○	○	○	●	○	essential

Rensa markering

Does your paper address subitem 20-i? *

Copy and paste relevant sections from the manuscript (include quotes in quotation marks "like this" to indicate direct quotes from your manuscript), or elaborate on this item by providing additional information not in the ms, or briefly explain why the item is not applicable/relevant for your study

"On the basis of these data, it is impossible to rule out whether the lack of time-by-treatment effect at follow-up is a result of a power issue or whether the 2 treatment conditions are equally effective in reducing insomnia symptoms in the long term. Given that 78% (42/54) of the sample had a sleeping problem duration of >5 years, explanations such as regression to the mean or spontaneous recovery are less likely."; "However, differences in therapist support may exist, although the same therapists provided both treatments. Moreover, the participants in the control condition completed fewer treatment modules, which may constitute a major limitation. In addition, because blinding is problematic in this research field, it is also plausible to assume that there is a substantial risk of contamination between both treatment arms. Finally, the results from the 6-month follow-up were rather uncertain, as the response rate declined considerably."

21) Generalisability (external validity, applicability) of the trial findings

NPT: External validity of the trial findings according to the intervention, comparators, patients, and care providers or centers involved in the trial

21-i) Generalizability to other populations

Generalizability to other populations: In particular, discuss generalizability to a general Internet population, outside of a RCT setting, and general patient population, including applicability of the study results for other organizations

	1	2	3	4	5	
subitem not at all important	○	○	○	○	○	essential

Does your paper address subitem 21-i?

Copy and paste relevant sections from the manuscript (include quotes in quotation marks "like this" to indicate direct quotes from your manuscript), or elaborate on this item by providing additional information not in the ms, or briefly explain why the item is not applicable/relevant for your study

Ditt svar

21-ii) Discuss if there were elements in the RCT that would be different in a routine application setting

Discuss if there were elements in the RCT that would be different in a routine application setting (e.g., prompts/reminders, more human involvement, training sessions or other co-interventions) and what impact the omission of these elements could have on use, adoption, or outcomes if the intervention is applied outside of a RCT setting.

	1	2	3	4	5	
subitem not at all important	○	○	○	○	○	essential

Does your paper address subitem 21-ii?

Copy and paste relevant sections from the manuscript (include quotes in quotation marks "like this" to indicate direct quotes from your manuscript), or elaborate on this item by providing additional information not in the ms, or briefly explain why the item is not applicable/relevant for your study

Ditt svar

OTHER INFORMATION

23) Registration number and name of trial registry

Does your paper address CONSORT subitem 23? *

Copy and paste relevant sections from the manuscript (include quotes in quotation marks "like this" to indicate direct quotes from your manuscript), or elaborate on this item by providing additional information not in the ms, or briefly explain why the item is not applicable/relevant for your study

"NCT03425942" Internet Administered CBT for Insomnia Comorbid With Chronic Pain (SoV-studien)

24) Where the full trial protocol can be accessed, if available

Does your paper address CONSORT subitem 24? *

Cite a Multimedia Appendix, other reference, or copy and paste relevant sections from the manuscript (include quotes in quotation marks "like this" to indicate direct quotes from your manuscript), or elaborate on this item by providing additional information not in the ms, or briefly explain why the item is not applicable/relevant for your study

https://clinicaltrials.gov/ct2/show/NCT03425942

25) Sources of funding and other support (such as supply of drugs), role of funders

Does your paper address CONSORT subitem 25? *

Copy and paste relevant sections from the manuscript (include quotes in quotation marks "like this" to indicate direct quotes from your manuscript), or elaborate on this item by providing additional information not in the ms, or briefly explain why the item is not applicable/relevant for your study

This study receives no funding."

X27) Conflicts of Interest (not a CONSORT item)

X27-i) State the relation of the study team towards the system being evaluated

In addition to the usual declaration of interests (financial or otherwise), also state the relation of the study team towards the system being evaluated, i.e., state if the authors/evaluators are distinct from or identical with the developers/sponsors of the intervention.

	1	2	3	4	5	
subitem not at all important	(●)	( )	( )	( )	( )	essential

Rensa markering

Does your paper address subitem X27-i?

Copy and paste relevant sections from the manuscript (include quotes in quotation marks "like this" to indicate direct quotes from your manuscript), or elaborate on this item by providing additional information not in the ms, or briefly explain why the item is not applicable/relevant for your study

Not applicable, are the same

About the CONSORT EHEALTH checklist

As a result of using this checklist, did you make changes in your manuscript? *

- ( ) yes, major changes
- ( ) yes, minor changes
- (●) no

What were the most important changes you made as a result of using this checklist?

Ditt svar

How much time did you spend on going through the checklist INCLUDING making changes in your manuscript *

8 hours

As a result of using this checklist, do you think your manuscript has improved? *

- ○ yes
- ● no
- ○ Övrigt:

Would you like to become involved in the CONSORT EHEALTH group?

This would involve for example becoming involved in participating in a workshop and writing an "Explanation and Elaboration" document

- ○ yes
- ◉ no
- ○ Övrigt:

Rensa markering

Any other comments or questions on CONSORT EHEALTH

Ditt svar

STOP - Save this form as PDF before you click submit

To generate a record that you filled in this form, we recommend to generate a PDF of this page (on a Mac, simply select "print" and then select "print as PDF") before you submit it.

When you submit your (revised) paper to JMIR, please upload the PDF as supplementary file.

Don't worry if some text in the textboxes is cut off, as we still have the complete information in our database. Thank you!

Final step: Click submit !

Click submit so we have your answers in our database!

Skicka

Rensa formuläret

Skicka aldrig lösenord med Google Formulär

Det här innehållet har varken skapats eller godkänts av Google. Anmäl otillåten användning - Användarvillkor - Integritetspolicy

Google Formulär
