# Supplementary material for: Internet-Delivered Cognitive Behavioral Therapy for Insomnia Comorbid With Chronic Pain: Randomized Controlled Trial
Source: J Med Internet Res. 2022 Apr 29;24(4):e29258. doi: 10.2196/29258 (PMC9107050; doi:10.2196/29258)
Supplement: Multimedia Appendix 2 [file jmir_v24i4e29258_app2.docx]

**Supplementary Material**

Wiklund et al. 2021. Internet-Delivered Cognitive Behavioral Therapy for Insomnia

Comorbid With Chronic Pain: Randomized Controlled Trial

Contents

[Supplementary Table 1. Diagnostic criteria for Insomnia Disorder according to DSM-V 2](#_Toc77593048)

[Supplementary Table 2. Inclusion and exclusion criteria 4](#_Toc77593049)

[Supplementary Table 3. Treatment content week-by-week 5](#_Toc77593050)

[Supplementary Table 4. Drop-out analysis 6](#_Toc77593051)

[Supplementary Table 5. Mixed models for ISI and weekly sleep diary scores from post to six-month follow-up (FU) 7](#_Toc77593052)

[Supplementary Table 6. Means, SDs, and changes in ISI and secondary outcomes at post and six-month FU for ICBT-i and AR groups 8](#_Toc77593053)

[Supplementary Table 7. Mixed models for secondary outcome scores (except for sleep diary) from post to six-month follow-up (FU) 10](#_Toc77593054)

[Supplementary Table 8. Patient Evaluation of ICBT-i and AR groups at various time points 11](#_Toc77593055)

[Supplementary Table 9. Results of Negative Effects Questionnaire (NEQ) of ICBT-i and AR groups 12](#_Toc77593056)

[Supplementary Figure 1. Mean average weekly KSS scores for the ICBT-I (1) and AR (0) groups from the beginning of the treatment (T1) to post (T5) 13](#_Toc77593057)

# Table S1. Diagnostic criteria for Insomnia Disorder according to DSM-V

| **A.** A predominant complaint of dissatisfaction with sleep quantity or quality, associated with one (or more) of the following symptoms:   1. Difficulty initiating sleep. (In children, this may manifest as difficulty initiating sleep without caregiver intervention.) 2. Difficulty maintaining sleep, characterized by frequent awakenings or problems returning to sleep after awakenings. (In children, this may manifest as difficulty returning to sleep without caregiver intervention.) 3. Early-morning awakening with inability to return to sleep. |
| --- |
| **B.** The sleep disturbance causes clinically significant distress or impairment in social, occupational, educational, academic, behavioral, or other important areas of functioning. |
| **C.** The sleep difficulty occurs at least 3 nights per week. |
| **D.** The sleep difficulty is present for at least 3 months. |
| **E.** The sleep difficulty occurs despite adequate opportunity for sleep. |
| **F.** The insomnia is not better explained by and does not occur exclusively during the course of another sleep-wake disorder (e.g., narcolepsy, a breathing-related sleep disorder, a circadian rhythm sleep-wake disorder, a parasomnia). |
| **H.** Coexisting mental disorders and medical conditions do not adequately explain the predominant complaint of insomnia. |
| **G.** The insomnia is not attributable to the physiological effects of a substance (e.g., a drug of abuse, a medication). |
| *Specify* if:   - With nonsleep disorder mental comorbidity, including substance use disorders - With other medical comorbidity - With other sleep disorder - Coding note: The code 780.52 (G47.00) applies to all three specifiers. Code also the relevant associated mental disorder, medical condition, or other sleep disorder immediately after the code for insomnia disorder in order to indicate the association.   *Specify* if:   - Episodic: Symptoms last at least 1 month but less than 3 months. - Persistent: Symptoms last 3 months or longer. - Recurrent: Two (or more) episodes within the space of 1 year.   Note: Acute and short-term insomnia (i.e., symptoms lasting less than 3 months but otherwise meeting all criteria with regard to frequency, intensity, distress, and/or impairment) should be coded as another specified insomnia disorder |
|  |

# Table S2. Inclusion and exclusion criteria

| **Inclusion criteria** | **Exclusion criteria** |
| --- | --- |
| Insomnia Severity Index (ISI) score > 14 | Shift worker or employed as a driver, operator of dangerous equipment, etc. |
| Chronic pain (i.e., pain lasting more than 3 months) | Sleep apnoea |
| Age 18-65 years | Restless legs syndrome |
| Able to set aside two hours a week for five weeks for treatment | Narcolepsy |
|  | Delayed sleep phase syndrome |
|  | Myalgic Encephalomyelitis/Chronic Fatigue Syndrome (ME/CFS) |
|  | Acute suicidality |
|  | Bipolar disorder |
|  | Psychotic disorders |
|  | Ongoing substance abuse |
|  | Received CBT for insomnia during the latest year |
|  | Pregnancy |
|  | Severe somatic disorder (e.g., ongoing cancer, severe neurological condition, insufficiently treated cardiac condition) |
|  | Impaired movement ability to such a degree that going to bed or getting out of bed requires assistance |
|  | Unable to read or wright in Swedish |
|  | Not having an internet-connected computer, cellular phone, or tablet |

# Table S3. Treatment content week-by-week

| Week | ICBT-i | Applied relaxation |
| --- | --- | --- |
| 1 | Rationale & sleep diary | Rationale, sleep diary, progressive relaxation & diaphragmatic breathing |
| 2 | Sleep restriction | Short progressive relaxation |
| 3 | Stimulus control | Conditioned relaxation |
| 4 | Activity balance | Differentiated relaxation |
| 5 | Relapse prevention | Quick relaxation |

# Table S4. Drop-out analysis

| **Characteristics;** n (%),  unless otherwise stated |  | **Completers (N=47)** |  | **Drop-outs**  **(N =7)** | **P value** |
| --- | --- | --- | --- | --- | --- |
| Age, years; mean (SD) |  | 48.6 (11.9) |  | 53.3 (14.8) | 0.35 |
| *Sex* |  |  |  |  | 0.20 |
| Men |  | 9 (19) |  | 0 (0) |  |
| Women |  | 38 (81) |  | 7 (100) |  |
| BMI |  | 25.8 (5.6) |  | 26.9 (3.4) | 0.59 |
| *Educational level* |  |  |  |  | 0.31 |
| Elementary school |  | 1 (2) |  | 0 (0) |  |
| Secondary school or vocational training |  | 26 (55) |  | 5 (71) |  |
| College or university |  | 20 (43) |  | 2 (29) |  |
| ISI; mean (SD) |  | 20.9 (2.92) |  | 21.1 (4.4) | 0.85 |
| GAD-7; mean (SD) |  | 7.5 (4.4) |  | 9.7 (6.8) | 0.25 |
| PHQ-9; mean (SD) |  | 13.6 (4.8) |  | 16.0 (6.4) | 0.24 |
| Pain Intensity NRS; mean (SD) |  | 6.6 (1.69) |  | 6.8 (1.8) | 0.77 |
| NPR; mean (SD) |  | 14.6 (9.7) |  | 14.4 (11.9) | 0.97 |
| PDI; mean (SD) |  | 41.9 (10.3) |  | 31.6 (12.2) | *0.02* |
| EQ5-VAS; mean (SD) |  | 42.9 (16.8) |  | 48.4 (20.4) | 0.43 |
| Duration of pain problems more than 5 years |  | 30 (64) |  | 4 (57) | 0.93 |
| Duration of sleep problems more than 5 years |  | 37 (79) |  | 5 (71) | 0.92 |

Notes: ICBT-i=Cognitive Behavioural Therapy for Insomnia; RLX=Applied Relaxation; BMI=Body Mass Index; ISI=Insomnia Severity Index; GAD-7=Generalised Anxiety Disorder 7-item; PHQ-9=The Patient Health Questionnaire 9-item; NRS=Numeric Rating Scale (NRS); NPR=Number of Pain Regions; PDI=Pain Disability Index; EQ5-VAS=EuroQol-5 Dimension Visual Analog Scale.

# Table S5. Mixed models for ISI and weekly sleep diary scores from post (W5) to six-month follow-up (FU)

|  | Estimate | SE | p | 95% CI |
| --- | --- | --- | --- | --- |
| **ISI** | | | | |
| Intercept (AIC:729.7) | 15.19 | 1.27 | **<0.001** | 12.7 to 17.6 |
| Treatment | −1.76 | 1.67 | 0.29 | −5.03 to 1.51 |
| Time (FU) | −0.07 | 0.25 | 0.77 | −0.56 to 0.42 |
| Time (FU)* Treatment | 0.18 | 0.30 | 0.53 | −0.39 to 0.77 |
| **SOL** | | | | |
| Intercept (AIC:608.3) | 47.72 | 9.75 | **<0.001** | 28.6 to 66.8 |
| Treatment | −19.12 | 12.71 | 0.14 | −44.0 to 5.79 |
| Time (FU) | −5.42 | 13.93 | 0.69 | −32.7 to 21.9 |
| Time (FU)*Treatment | −4.10 | 17.45 | 0.81 | −38.3 to 30.1 |
| **WASO** | | | | |
| Intercept (AIC:595) | 63.38 | 9.68 | **<0.001** | 44.4 to 82.3 |
| Treatment | −22.94 | 12.78 | 0.08 | −47.9 to 2.09 |
| Time (FU) | 0.65 | 6.59 | 0.92 | −12.2 to 13.5 |
| Time (FU)*Treatment | 10.52 | 8.38 | 0.22 | −5.91 to 26.9 |
| **TST** | | | | |
| Intercept (595.3) | 336.4 | 21.4 | **<0.001** | 318.4 to 402.3 |
| Treatment | −11.2 | 28.3 | 0.69 | −66.5 to 44.2 |
| Time (FU) | −17.4 | 11.7 | 0.14 | −40.3 to 5.52 |
| Time (FU)*Treatment | 28 | 14.9 | 0.07 | −1.23 to 57.2 |
| **SE** | | | | |
| Intercept (AIC:458.9) | 67.38 | 3.45 | **<0.001** | 60.6 to 74.1 |
| Treatment | 8.98 | 4.55 | 0.07 | 0.06 to 17.9 |
| Time (FU) | −1.44 | 3.01 | 0.64 | −7.34 to 4.47 |
| Time (FU)*Treatment | −2.66 | 3.85 | 0.49 | −10.2 to 4.88 |
| **EMA** | | | | |
| Intercept (AIC:573) | 7.14 | 0.37 | **<0.001** | 6.41 to 7.88 |
| Treatment | −0.44 | 0.49 | 0.38 | −1.41 to 0.53 |
| Time (FU) | −0.65 | 0.32 | **0.05** | −1.27 to −0.03 |
| Time (FU)*Treatment | 0.35 | 0.41 | 0.38 | −0.44 to 1.15 |

Notes: ISI=Insomnia Severity Index; SE=Standard Error; CI=Confidence Interval; SOL=Sleep Onset Latency; WASO= Wake Time after Sleep Onset; TST=Total Sleep Time; SE=Sleep Efficiency; EMA=Early Morning Awakenings; KSS= Karolinska Sleepiness Scale; FU=Follow-up.

# Table S6. Means, SDs, and changes in ISI and secondary outcomes at post (W5) and six-month FU for ICBT-i and AR groups

|  | Post (W5) | | |  | Change (pre-post) | | | 6 m FU | | |  | Change (pre-6m FU) | |
| --- | --- | --- | --- | --- | --- | --- | --- | --- | --- | --- | --- | --- | --- |
| Outcome | M | SD | N |  | M diff | SD |  | M | SD | N |  | M diff | SD |
| ISI |  |  |  |  |  |  |  |  |  |  |  |  |  |
| ICBT-i | 12.7 | 5.8 | 27 |  | 8.4 | 4.7*** |  | 13.6 | 5.7 | 23 |  | 6.7 | 5.4*** |
| AR | 15.5 | 5.8 | 20 |  | 5.0 | 5.4*** |  | 14.2 | 5.8 | 15 |  | 6.1 | 5.2*** |
| GAD-7 |  |  |  |  |  |  |  |  |  |  |  |  |  |
| ICBT-i | 5.2 | 3.4 | 27 |  | 1.7 | 3.6* |  | 5.0 | 2.2 | 23 |  | 1.6 | 3.7* |
| AR | 6.0 | 5.1 | 20 |  | 1.3 | 5.6^ns^ |  | 3.3 | 2.5 | 14 |  | 3.5 | 4.4* |
| PHQ-9 |  |  |  |  |  |  |  |  |  |  |  |  |  |
| ICBT-i | 9.4 | 5.1 | 27 |  | 3.37 | 4.1*** |  | 9.1 | 3.6 | 23 |  | 3.9 | 4.1*** |
| AR | 10.2 | 5.8 | 20 |  | 3.65 | 6.5* |  | 7.4 | 3.4 | 15 |  | 6.3 | 5.1*** |
| Pain Intensity NRS |  |  |  |  |  |  |  |  |  |  |  |  |  |
| ICBT-i | 6.2 | 1.4 | 27 |  | 0.14 | 1.4^ns^ |  | 5.7 | 1.9 | 23 |  | 0.34 | 1.9^ns^ |
| AR | 6.7 | 1.9 | 20 |  | 0.04 | 1.1^ns^ |  | 5.7 | 1.9 | 15 |  | 1.06 | 1.3** |
| NPR |  |  |  |  |  |  |  |  |  |  |  |  |  |
| ICBT-i | 12.5 | 8.4 | 27 |  | -0.14 | 2.9^ns^ |  | 12.2 | 8.6 | 23 |  | 0.56 | 4.6^ns^ |
| AR | 15.1 | 8.5 | 20 |  | 1.6 | 5.5^ns^ |  | 12.6 | 9.2 | 15 |  | 3.4 | 7.4^ns^ |
| PDI |  |  |  |  |  |  |  |  |  |  |  |  |  |
| ICBT-i | 36.8 | 11.9 | 27 |  | 3.4 | 9.5^ns^ |  | 38.1 | 13.6 | 23 |  | 2.8 | 12.1^ns^ |
| AR | 39.5 | 13.4 | 20 |  | 2.8 | 9.3^ns^ |  | 35.2 | 9.1 | 14 |  | 5.8 | 10.1* |
| EQ5-VAS |  |  |  |  |  |  |  |  |  |  |  |  |  |
| ICBT-i | 50.4 | 17.7 | 27 |  | 6.4 | 16.8^ns^ |  | 49.7 | 19.5 | 23 |  | 8.5 | 18.7* |
| AR | 46.7 | 17.8 | 20 |  | 2.9 | 15.3^ns^ |  | 53.0 | 17.1 | 14 |  | 7.6 | 18.2^ns^ |

Notes: ICBT-i=Cognitive Behavioural Therapy for Insomnia; AR=Applied Relaxation; ISI= Insomnia Severity Index; GAD-7= Generalised Anxiety Disorder 7-item; PHQ-9= The Patient Health Questionnaire 9-item; NRS=Numeric Rating Scale (NRS); NPR=Number of Pain Regions; PDI=Pain Disability Index; EQ5-VAS= EuroQol-5 Dimension Visual Analog Scale.

# Table S7. Mixed models for secondary outcome scores (except for sleep diary) from post (W5) to six-month follow-up (FU)

| **Outcomes** | **Estimate** | **SE** | **p** | **95% CI** |
| --- | --- | --- | --- | --- |
| GAD-7 | | | | |
| Intercept (AIC:447.2) | 6.05 | 0.81 | **<0.001** | 4.45 to 7.65 |
| Treatment | −0.82 | 1.07 | 0.44 | −2.93 to 1.28 |
| Time (FU) | −2.19 | 0.84 | **0.01** | −3.84 to −0.53 |
| Time (FU) * Treatment | 2.02 | 1.08 | 0.07 | −0.09 to 4.14 |
| PHQ-9 | | | | |
| Intercept (AIC:496.3) | 10.25 | 1.07 | **<0.001** | 8.16 to 12.33 |
| Treatment | −0.84 | 1.41 | 0.55 | −3.59 to 1.91 |
| Time (FU) | −2.53 | 1.05 | **0.02** | −4.59 to −0.47 |
| Time (FU) * Treatment | 2.52 | 1.36 | 0.07 | −0.14 to 5.18 |
| Pain Intensity NRS | | | | |
| Intercept (AIC:339.5) | 6.70 | 0.40 | **<0.001** | 5.91 to 7.48 |
| Treatment | −0.52 | 0.53 | 0.33 | −1.55 to 0.53 |
| Time (FU) | −0.76 | 0.46 | 0.11 | −1.66 to 0.14 |
| Time (FU) * Treatment | 0.44 | 0.59 | 0.46 | −0.72 to 1.61 |
|  | NPR |  |  |  |
| Intercept (AIC:542.9) | 15.15 | 1.91 | **<0.001** | 11.4 to 18.8 |
| Treatment | −2.63 | 2.52 | 0.30 | −7.57 to 2.30 |
| Time (FU) | −1.73 | 0.88 | 0.06 | −3.46 to 0.00 |
| Time (FU) * Treatment | 1.23 | 1.13 | 0.28 | −0.99 to 3.45 |
| PDI | | | | |
| Intercept (650.9) | 39.55 | 2.81 | **<0.001** | 34.0 to 45.0 |
| Treatment | −2.70 | 3.71 | 0.47 | −9.96 to 4.56 |
| Time (FU) | −4.40 | 2.73 | 0.11 | −9.73 to 0.94 |
| Time (FU) * Treatment | 5.85 | 3.48 | 0.10 | −0.96 to 12.66 |
| EQ5-VAS | | | | |
| Intercept (AIC:733.1) | 46.70 | 4.06 | **<0.001** | 38.7 to 54.6 |
| Treatment | 3.78 | 5.36 | 0.48 | −6.72 to 14.2 |
| Time (FU) | 6.27 | 6.08 | 0.31 | −5.66 to 18.2 |
| Time (FU) * Treatment | −6.80 | 7.83 | 0.39 | −22.1 to 8.56 |

Notes: GAD-7=Generalised Anxiety Disorder 7-item; PHQ-9=The Patient Health Questionnaire 9-item; NRS=Numeric Rating Scale (NRS); NPR=Number of Pain Regions; PDI=Pain Disability Index; EQ5-VAS= EuroQol-5 Dimension Visual Analog Scale; SE=Standard Error; CI=Confidence Interval.

# Table S8. Patient Evaluation of ICBT-i and AR groups at various time points

|  | Questions | | |  |
| --- | --- | --- | --- | --- |
|  | Q1: To what extent do you think this treatment will be helpful in reducing your sleep problems (median; IQR) | Q2: How likely is it that you would recommend this treatment to a relative/friend with the same kind of problem (median; IQR) | Q3: To what extent do you think this treatment has been helpful in reducing your sleep problems? (median; IQR) | Difference between groups |
| ICBT-i |  |  |  |  |
| W2 (week 2) | 4 (3­­–4) | - | - | 0.74 |
| Post (week 5) | - | 4 (3–6) | - | 0.78 |
| 6-mo. FU | - | 5 (3–6) | - | 0.59 |
| 6-mo. FU | - | - | 4 (3–5) | 0.07 |
| AR |  |  |  |  |
| W2 (week 2) | 3.5 (3–4) | - | - | 0.74 |
| Post (week 5) | - | 5 (3.5–6) | - | 0.78 |
| 6-mo. FU | - | 4 (3–6) | - | 0.59 |
| 6 mo. FU | - | - | 2 (2–4) | 0.07 |

Notes: ICBT-i=Cognitive Behavioural Therapy for Insomnia; AR=Applied Relaxation; 6-mo. FU=Six-month Follow-up.

# Table S9. Results of Negative Effects Questionnaire (NEQ) of ICBT-i and AR groups

| **NEQ** | **ICBT-i** | | **AR** | |
| --- | --- | --- | --- | --- |
|  | Yes (n, %) | Negative effect  M (SD) | Yes (n, %) | Negative effect  M (SD) |
| I had bigger problems with my sleep | 6 (22) | 0.83 (0.98) | 3 (15) | 2.33 (0.57) |
| I felt more stressed | 13 (48)* | 1.61 (0.76) | 10 (50)*** | 1.90 (0.74) |
| I experienced more anxiety | 2 (7) | 1.5 (0.70) | 4 (20) | 2.00 (0.81) |
| I became more worried | 2 (7) | 1 (0) | 4 (20) | 1.75 (0.50) |
| I felt more depressed | 4(14) | 0 (0) | 5 (25) | 1.80 (0.44) |
| I experienced greater hopelessness | 3(11) | 0.33 (0.57) | 3 (15) | 1.66 (0.58) |
| I got worse self-esteem | 0 (0) | - | 1 (5) | 2 (-) |
| I lost faith in myself | 0 (0) | - | 1 (5) | 1 (-) |
| I felt sadder | 2 (7) | 0 (0) | 4 (20) | 2 (0) |
| I perceived myself as less competent | 0 (0) | - | 2 (10) | 2 (0) |
| I experienced more unpleasant feelings | 2 (7) | 0 (0) | 3 (15) | 1.66 (0.58) |
| I felt that what I sought help for worsened | 1 (4) | 1 (0) | 2 (10) | 1.50 (0.70) |
| I experienced that old unpleasant memories were brought to life | 4(14) | 0.50 (0.57) | 3 (15) | 1.33 (0.58) |
| I was afraid that other people would understand that I was going for a treatment | 0 (0) | - | 0 (0) | - |
| I got thoughts that it would be better if I was no longer there or that I should take my own life | 1 (4) | 0 (0) | 0 (0) | - |
| I began to feel ashamed in front of others that I was undergoing treatment | 0 (0) | - | 0 (0) | - |
| I stopped believing that things could get better | 3 (11) | 0.66 (0.57) | 6 (30) | 1.66 (0.52) |
| I began to think that what I was looking for help for could not be influenced for the better | 6 (22) | 0.66 (0.51) | 3 (15) | 2.33 (0.57) |
| I stopped believing that there is help available | 5(18) | 0.4 (0.54) | 8 (40) | 1.87 (0.83) |
| I think I have developed an addiction to my treatment | 0 (0) | - | 0 (0) | - |
| I seem to have developed an addiction to my therapist | 0 (0) | - | 0 (0) | - |
| I did not always understand my treatment | 6 (22) | 1 (0) | 1 (5) | 2 (-) |
| I did not always understand my therapist | 5 (18) | 1 (0) | 0 (0) | - |
| I did not trust my treatment | 2 (7) | 0.50 (0.70) | 0 (0) | - |
| I did not trust my therapist | 0 (0) | - | 0 (0) | - |
| I felt that the treatment did not give any results | 8 (29) | 0.62 (0.51) | 6 (30) | 2.16 (0.75) |
| I did not feel that my expectations of the treatment were met | 7 (25) | 1 (0) | 3 (15) | 2 (-) |
| I did not feel that my expectations of the therapist were met | 1 (4) | 1 (-) | 1 (5) | 2 (-) |
| I felt that the quality of the treatment was poor | 2 (7) | 0.50 (0.70) | 0 (0) | - |
| I felt that the treatment did not suit me | 10 (37) ** | 0.90 (0.31) | 3 (15) | 1.33 (0.57) |
| I felt that I did not get any closer relationship with my therapist | 2 (7) | 0.50 (0.70) | 1 (5) | 0(-) |
| I found the treatment unmotivating | 2 (7) | 0.50 (0.70) | 0 (0) | - |

Notes: ICBT-i=Cognitive Behavioural Therapy for Insomnia; AR=Applied Relaxation

*8 out of 13 who felt more stressed probably caused by the treatment itself.

** 9 out of 10 who felt that the treatment did not suit them probably caused by the treatment itself.

***5 out of 10 who felt more stressed probably caused by the treatment itself.

**** 4 out of 8 who stopped believing that there is help available probably caused by the treatment itself.

t-test showed no statistically significant differences between treatment and control group.

# Figure S1. Mean average weekly KSS scores for the ICBT-I (1) and AR (0) groups from the beginning of the treatment (W1) to post (W5).


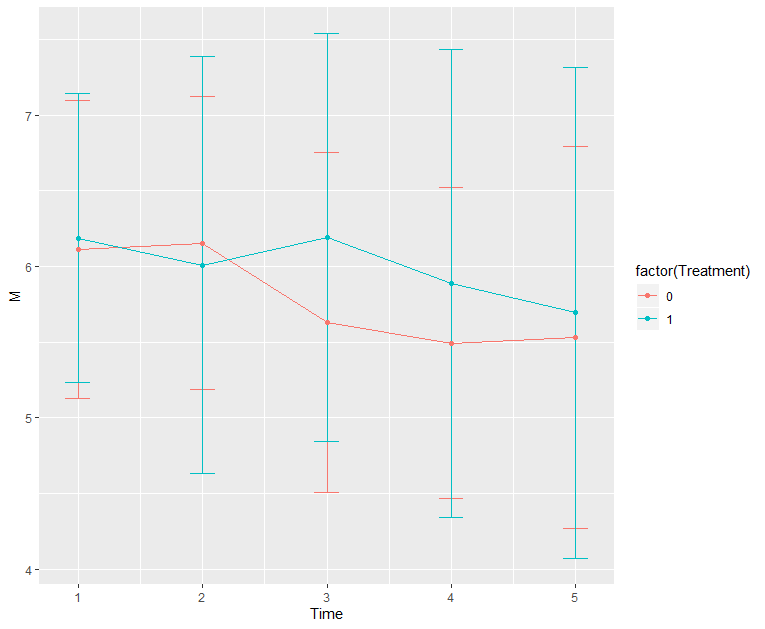


Notes: 1=week 1, 2=week 2, 3=week 3, 4=week 4, 5=week 5 (post)
